# Diagnostic performance of β-(1→3)-D-glucan, two *Candida* antigen, and five anti-*Candida* antibody assays in ICU patients with sepsis and high risk for invasive candidiasis: a secondary endpoint of the CandiSep randomized clinical trial

**DOI:** 10.1128/jcm.01500-25

**Published:** 2026-04-23

**Authors:** Lea Standl, Timo Huber, Frank Bloos, Daniel Thomas-Rüddel, Johannes Träger, Stefan Kluge, Falk Fichtner, Philipp Simon, Klaus Kogelmann, Geraldine de Heer, Sven‑Olaf Kuhn, Dominik Jarczak, Johann Motsch, Gunther Hempel, Norbert Weiler, Andreas Weyland, Matthias Drüner, Matthias Gründling, Patrick Meybohm, Daniel Richter, Onnen Moerer, Ulf Günther, Dirk Schädler, Alexander Zarbock, Christian Putensen, Ixchel Castellanos, Oliver Kurzai, Peter Schlattmann, Oliver A. Cornely, Michael Bauer, Thomas Lehmann, Giuseppe Valenza, Christian Bogdan, Jürgen Held, on behalf of the SepNet Critical Care Trials Group

**Affiliations:** 1 Mikrobiologisches Institut - Klinische Mikrobiologie, Immunologie und Hygiene, Universitätsklinikum Erlangen und Friedrich-Alexander-Universität (FAU), Erlangen, Germany; 2 Department of Anesthesiology, University Hospital Erlangen 27168 , Erlangen, Germany; 3 Center for Sepsis Control and Care, Jena University Hospital 553346, Jena, Germany; 4 Department of Anesthesiology and Intensive Care Medicine, Jena University Hospital 39065, Jena, Germany; 5 Department of Anesthesiology, Montefiore Medical Center and Albert Einstein College of Medicine, Bronx, New York, USA; 6 Department of Intensive Care Medicine, University Hospital Hamburg-Eppendorf, Hamburg, Germany; 7 Department of Anesthesiology and Intensive Care Medicine, University of Leipzig Medical Centre, Leipzig, Germany; 8 Anesthesiology and Operative Intensive Care, Faculty of Medicine, University of Augsburg 531257, Augsburg, Germany; 9 Department of Anaesthesiology, Klinikum Leer 39835 , Leer, Germany; 10 Department of Anesthesiology and Intensive Care Medicine, University Hospital Greifswald, Greifswald, Germany; 11 Department of Anesthesiology, University Hospital Heidelberg 27178, Heidelberg, Germany; 12 Department of Anesthesiology and Intensive Care Medicine, University Hospital Schleswig-Holstein, Campus Kiel 15056, Kiel, Germany; 13 Research Center Neurosensory Science, Carl von Ossietzky Universität Oldenburg 11233, Oldenburg, Germany; 14 Department of Anesthesiology and Intensive Care Medicine, Hospital Emden, Emden, Germany; 15 Department of Anaesthesiology, Intensive Care, Emergency and Pain Medicine, University Hospital Würzburg 27207, Würzburg, Germany; 16 Department of Anesthesiology, University Medical Center, Georg-August-University 27177, Göttingen, Germany; 17 University Clinic of Anaesthesiology, Intensive Care, Emergency Medicine and Pain Therapy, Klinikum Oldenburg 37099, Oldenburg, Germany; 18 Department Of Adult Intensive Care, University Medical Center Groningen, Groningen, the Netherlands; 19 Department of Anesthesiology, Intensive Care and Pain Medicine, University Hospital Münster 235721, Münster, Germany; 20 Division of Intensive Care Medicine, Dept. of Anesthesiology and Intensive Care Medicine, University Hospital Bonn 39062, Bonn, Germany; 21 Institute for Hygiene and Microbiology, Julius Maximilians University Würzburg 9190, Würzburg, Germany; 22 National Reference Center for Invasive Fungal Infections NRZMyk, Leibniz Institute for Natural Product Research and Infection Biology – Hans-Knoell-Institute 28406, Jena, Germany; 23 Computer Sciences and Data Science, Institute of Medical Statistics, Jena University Hospital 39065, Jena, Germany; 24 Department I of Internal Medicine, Excellence Center for Medical Mycology (ECMM), Faculty of Medicine and University Hospital Cologne, University of Cologne 61059, Cologne, Germany; 25 Institute of Translational Research, Faculty of Medicine and University Hospital Cologne, Cologne Excellence Cluster on Cellular Stress Responses in Aging-Associated Diseases (CECAD), University of Cologne, Cologne, Germany; 26 Faculty of Medicine and University Hospital Cologne, Clinical Trials Centre Cologne (ZKS Köln), University of Cologne 61059, Cologne, Germany; 27 German Centre for Infection Research (DZIF), Partner Site Bonn-Cologne, Cologne, Germany; 28 Center for Clinical Studies, Jena University Hospital 39065, Jena, Germany; 29 FAU Profile Center for Immunomedicine, Friedrich-Alexander-Universität (FAU) Erlangen-Nürnberg 9171, Erlangen, Germany; University of Calgary, Calgary, Alberta, Canada

**Keywords:** candidemia, bloodstream infection, colonization, combination, mannan, anti-mannan, beta-D-glucan, diagnosis

## Abstract

Invasive *Candida* infection (ICI) is the most common fungal infection in critically ill patients. This study analyzed the performance of various biomarkers in sera from the CandiSep trial, a randomized, multicenter trial including 342 sepsis patients at high risk for ICI across 18 German intensive care units. ICI and candidemia were diagnosed in 48 (14.0%) and 14 (4.1%) patients, respectively. Sera collected on 2 consecutive days at sepsis onset were analyzed for β-(1→3)-D-glucan (BDG; Fungitell), mannan (Platelia-*Candida*-Ag-Plus [Platelia-Mn] and Serion-ELISA-antigen-*Candida* [Serion-Mn]), and anti-*Candida* antibodies (Platelia-*Candida*-Ab-Plus, Serion-ELISA-*Candida albicans*-IgA/IgM/IgG, and Virclia-*Candida albicans*-germ-tube-antibody-IgG-Monotest). Only antigen levels (BDG, Platelia-Mn, Serion-Mn), but not anti-Candida antibody levels, were significantly elevated in ICI patients. Antigen levels were unaffected by *Candida* colonization, while antibody levels were significantly increased. Sensitivity and specificity at the manufacturer’s cutoffs were unsatisfactory. Performance improved by adjusting cutoffs: BDG required a 3-fold increase, while all others required lowering. At 80% specificity, sensitivities (95% confidence intervals) for the diagnosis of ICI and candidemia were as follows: BDG (cutoff >280 pg/mL), 46% (31.4–60.8) and 64% (35.1–87.2); Platelia-Mn (cutoff >50 pg/mL), 38% (24.0–52.6) and 64% (35.1–87.2); Serion-Mn (cutoff >0.7 U/mL), 38% (24.0–52.6) and 50% (23.0–77.0), and below 28% and 43% for all antibody assays. Area under the receiver operating characteristic curve comparisons showed no significant differences between antigen assays. Combining biomarkers, either simultaneously or sequentially, offered no diagnostic advantage over single-biomarker use. In conclusion, anti-*Candida* antibody assays are likely influenced by colonization and have limited diagnostic utility. Antigen assays offer similar diagnostic value but require cutoff optimization. Combining biomarkers did not enhance diagnostic yield in our cohort.

**IMPORTANCE:**

Our main findings based on our specific ICU cohort were as follows: (i) only *Candida* antigen levels, not anti-*Candida* antibody levels, were significantly elevated in ICI and candidemia. (ii) In patients without ICI, Candida colonization was not associated with altered antigen levels, but with elevated antibody levels. (iii) Sensitivity and specificity at the manufacturer’s cutoffs were unsatisfactory, and cutoffs should be significantly adjusted. Potential optimal cutoff values are proposed by us. (iv) The evaluated antigen assays demonstrated overall comparable diagnostic performance in this study. (v) Anti-*Candida* antibody assays did not provide a meaningful diagnostic contribution. (vi) The combination of biomarkers offered no diagnostic advantage over the use of individual biomarkers, neither simultaneously nor sequentially. Our results suggest that the use of a single *Candida* antigen test with a cohort-adapted cutoff value may be sufficient. Furthermore, avoiding biomarker combinations could potentially reduce healthcare costs. The clinical implications of these findings should be interpreted with caution, given the limited data with associated large confidence intervals for candidemia and the cohort-specific nature of the study. Our results should therefore be confirmed in larger studies and additionally with other patient groups.

## INTRODUCTION

Candidemia is the most common fungal bloodstream infection in critically ill patients in Europe and the USA (1, 2). Since bacteremia and candidemia are often clinically indistinguishable, only every second patient later diagnosed with candidemia receives an antifungal agent as empirical anti-infective therapy (3). The other half of the patients only receives antifungal therapy when blood cultures (BC) turn positive and show growth of yeasts, that is, with a delay of 1–2 days after the onset of symptoms (3). However, a delay in initiating appropriate antifungal treatment is associated with increased mortality (4, 5). A pre-emptive approach using rapid diagnostic tests might be a solution to shorten the time until effective therapy begins (5). For this purpose, assays for the detection of β-(1→3)-D-glucan (BDG), mannan (Mn), and anti-*Candida* antibodies (anti-*Candida*-Ab) in sera have become commercially available, with the results available within just a few hours (6). While BDG is produced by various medically relevant fungal genera, including *Candida*, Mn, and anti-*Candida*-Ab assays are largely *Candida*-specific (6, 7).

In the past, the diagnostic performance of *Candida* biomarkers has mostly been studied retrospectively in case-control studies. The sensitivities and specificities in these studies were ~75%–80% and ~80% for BDG, 58% and 93% for Mn, and 59% and 83% for anti-Mn-Ab, respectively (5, 6). Until now, direct comparisons between different assays for the detection of BDG, Mn, and anti-*Candida*-Ab, and prospective studies investigating the diagnostic performance of combinations of different biomarkers in ICU patients have been rare (8–14). Accordingly, current guidelines are cautious to recommend a pre-emptive approach, as there is a lack of convincing data demonstrating a beneficial effect (15). For this reason, several international experts in the field of infectious diseases and intensive care medicine have recently pointed out that the evaluation of strategies based on fungal biomarkers in intensive care units (ICUs) is both timely and necessary (16, 17). Therefore, the present study addressed the secondary objective of the CandiSep trial (18, 19), namely the analysis of several fungal biomarkers (BDG, Mn, and anti-*Candida*-Ab) with different assays alone or in combination in the sera of adult patients with sepsis and an increased risk for developing invasive *Candida* infection (ICI).

## MATERIALS AND METHODS

The CandiSep trial was a prospective, randomized, multicenter study comparing BDG-guided antifungal therapy versus standard of care in 342 adult patients with sepsis and increased risk for invasive *Candida* infection (total parenteral nutrition, abdominal surgery within 7 days, prior antimicrobial therapy >48 h, or prior renal replacement therapy) in 18 German ICUs. Details on the cohort are provided in the original CandiSep publications (18, 19). From each patient, a serum sample and concomitant BC were collected at randomization and 24 h after enrollment. In addition, microbiological samples were taken at baseline and after 7 and 14 days from the nose/throat, axilla, rectum/feces, urine, and tracheal/bronchial secretions. An ongoing or immediately planned systemic antifungal therapy was an exclusion criterion. Three patients withdrew consent and were excluded. ICI, consisting of deep-seated candidiasis (e.g., peritonitis, meningitis, and pleural empyema) and candidemia, was diagnosed at 96 h in 48 (14.2%) patients. Thirty-four patients (10.0%) had deep-seated candidiasis without candidemia, 4 (1.2%) patients had deep-seated candidiasis with candidemia, and 10 (2.9%) patients had candidemia without deep-seated candidiasis. For 14 patients, only the first serum sample was available due to early death. In total, 664 sera were analyzed. Testing was approved by the Ethics Committee of the University Hospital Jena; written consent was obtained from patients or representatives.

The sera were tested for BDG during the CandiSep trial (09/2016 to 09/2019) using the Fungitell assay (Associates of Cape Cod, USA) and were then immediately aliquoted and frozen at −80°C. In 2019, the sera were thawed and tested in batch for Mn with the Platelia Candida Ag Plus assay (Platelia-Mn; Bio-Rad, France) and the SERION ELISA *antigen* Candida (Serion-Mn; SERION Diagnostics, Germany), as well as for anti-*Candida*-Ab with the Platelia Candida Ab Plus assay (Platelia-Ab; Bio-Rad, France), the SERION ELISA *classic C. albicans* IgA/IgM/IgG assays (Serion-IgA/IgM/IgG; SERION Diagnostics, Germany) and the INVASIVE CANDIDIASIS (CAGTA) Virclia IgG Monotest (CAGTA; Vircell S.L., Spain). The samples underwent a maximum of one freeze–thaw cycle between BDG testing and batch testing. All assays were performed according to the manufacturer’s recommendations.

Diagnostic performance was calculated for (i) at least one positive serum, (ii) both sera positive, (iii) simultaneous, and (iv) sequential testing.

The Mann-Whitney U test was used for the comparison of continuous variables. Differences were considered significant for *P* < 0.05. Optimal cutoffs were determined by a receiver operating characteristic (ROC) analysis (maximum Youden index). Since this is a data-driven approach, we used bootstrapping to investigate the corresponding variability. Additionally, we performed decision curve analyses to evaluate the clinical usefulness of the respective diagnostic tests, that is, we plotted the “net benefit” across a range of threshold probabilities (20). Optimal biomarker combinations for simultaneous testing were identified by multiple binary logistic regression analyses using the forward Wald procedure. In this procedure, the binary dependent variable (ICI or candidemia) is predicted by a model of several independent variables (biomarkers). To create a prediction model that is as efficient as possible, biomarkers are stepwise included if they make a significant contribution to the prediction model. With a ROC analysis of the predicted probabilities, the area under the ROC curve (AUROC) can be calculated. Furthermore, internal validation was performed using bootstrap-based bias optimism correction (21). Calibration of the models with respect to key parameters was assessed using the Brier score. The optimal biomarker combinations for sequential testing were identified by decision tree analysis using Chi-squared Automatic Interaction Detection (CHAID). Statistical analysis was performed using R 4.4.1 (22) with package “cutpointr” (23),”dcurves” (24), “rms” (25), SPSS-V29 (IBM, USA), and MedCalc-V22 (MedCalc Software Ltd, Belgium).

## RESULTS

### Biomarker levels

**Fig 1:**
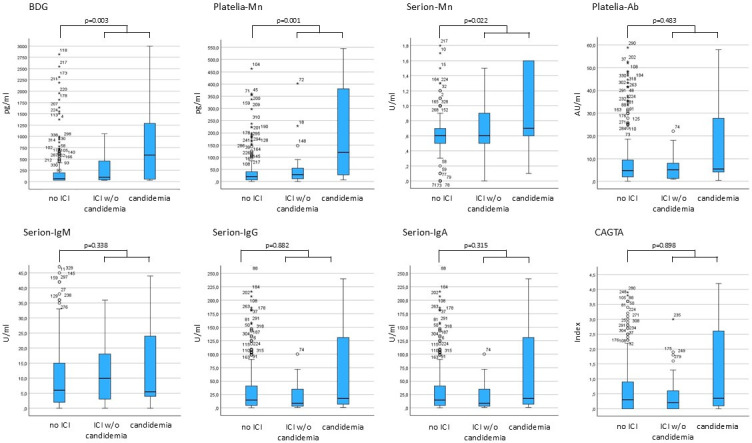
Boxplots showing the median biomarker levels of patients with and without ICI and candidemia (bold line, median; box, interquartile range (IQR); antenna, 1.5× the IQR; circles, outliers; stars, extreme outliers). The x-axis has been reduced to ensure better visualization of the box and the antennas. For this reason, not all outliers are shown. The *P*-value refers to the comparison of biomarker concentrations in patients with (deep-seated IC and candidemia) and without ICI. BDG, β-(1→3)-D-glucan; Mn, mannan; Ab, antibody; CAGTA, Candida albicans germ tube antibodies; U, units; AU, arbitrary units; ICI, invasive *Candida* infection; w/o, without.

The median biomarker levels of the first serum sample in patients with deep-seated candidiasis, candidemia, and without ICI are shown in Fig. 1 and Table S1. Only the antigen levels (BDG, Platelia-Mn, Serion-Mn), but not the anti-*Candida*-Ab levels, were significantly higher in patients with ICI compared to patients without ICI. Furthermore, patients with candidemia had higher BDG and Platelia-Mn levels compared to those with deep-seated candidiasis; however, the differences were not statistically significant (*P* = 0.05 and *P* = 0.06, respectively).

**Fig 2:**
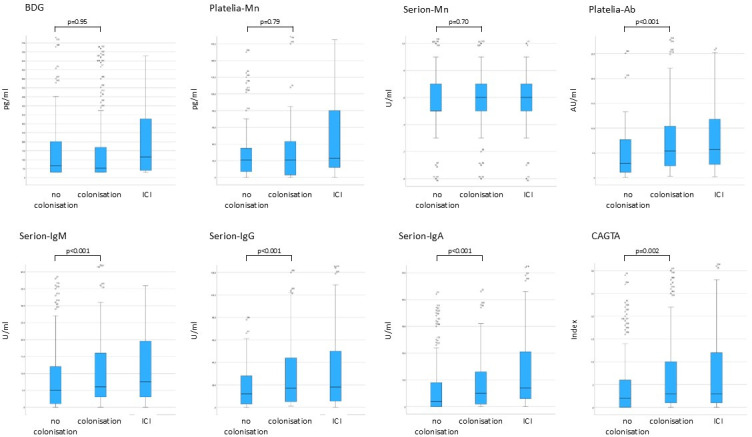
Boxplots showing the median biomarker levels of non-infected patients with and without *Candida* colonization and of patients with ICI (bold line, median; box, interquartile range (IQR); antenna, 1.5× the IQR; circles, outliers; stars, extreme outliers). The x-axis has been reduced to ensure better visualization of the box and the antennas. For this reason, not all outliers are shown. The *P*-value refers to the comparison of biomarker concentrations in patients with and without *Candida* colonization.

Interestingly, the median antigen levels were the same in patients with and without *Candida* colonization. In contrast, anti-*Candida*-Ab levels were significantly higher in patients with *Candida* colonization than in patients without colonization (*P* < 0.001 and *P* = 0.002, respectively), and the levels in colonized patients were as high as in patients with ICI (Fig. 2; Table S2).

### Diagnostic performance of single biomarkers at the manufacturer’s cutoff values

**Fig 3:**
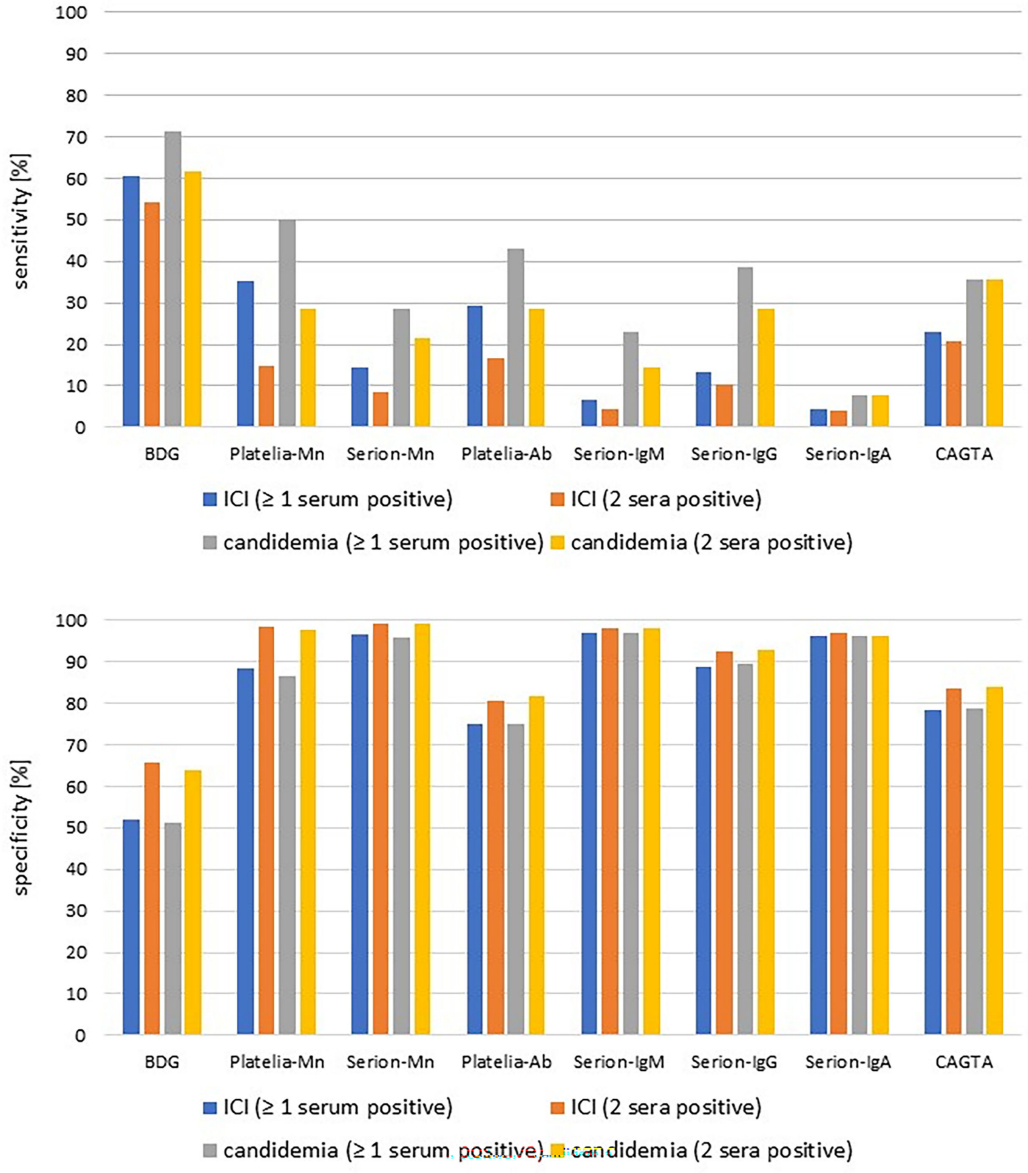
Sensitivity and specificity of individual biomarkers for positivity criteria 1 and 2. BDG, β-(1→3)-D-glucan; Mn, mannan; Ab, antibody; CAGTA, *Candida albicans* germ tube antibodies; ICI, invasive *Candida* infection.

The sensitivities, specificities, and positive and negative predictive values (PPV and NPV) for the positivity criteria 1 (at least one positive serum sample) and 2 (two positive serum samples) are shown in Table 1 and Fig. 3. PPV and NPV across different prevalences of ICI and candidemia are provided in Table S3.

**TABLE 1:** Diagnostic performance of biomarker assays at the manufacturer’s cutoff values for positivity criteria 1 and 2^*a*^

Positivity criteria	Biomarker	Invasive *candida* infection				Deep-seated candidiasis (w/o candidemia)				Candidemia			
		Sensitivity (%) (95% CI)	Specificity (%) (95% CI)	Ppv (%) (95% CI)	Npv (%) (95% CI)	Sensitivity (%) (95% CI)	Specificity (%) (95% CI)	Ppv (%) (95% CI)	Npv (%) (95% CI)	Sensitivity (%) (95% CI)	Specificity (%) (95% CI)	Ppv (%) (95% CI)	Npv (%) (95% CI)
≥ 1 serum sample positive	BDG	60.4 (45.0–74.0)	51.9 (46.0–57.8)	17.2 (13.8–21.2)	88.8 (84.6–92.0)	55.9 (37.9–72.8)	51.9 (46.0–57.8)	12.0 (9.0–15.8)	91.0 (87.2–93.7)	71.4 (41.9–91.6)	51.1 (45.5–56.6)	5.9 (4.2–8.1)	97.7 (94.8–99.0)
	Platelia-Mn	35.4 (22.2–50.5)	88.3 (84.1–91.8)	33.4 (23.4–45.2)	89.2 (87.0–91.1)	29.4 (15.1–47.5)	29.4 (15.1–47.5)	22.7 (13.8–35.1)	91.5 (89.6–93.0)	50.0 (23.0–77.0)	86.5 (82.3–90.0)	13.6 (8.0–22.2)	97.6 (96.0–98.6)
	Serion-Mn	14.6 (6.1–27.8)	96.6 (93.8–98.3)	41.3 (21.9–63.7)	87.2 (85.8–88.5)	8.8 (1.9–23.7)	96.6 (93.8–98.3)	23.1 (8.0–50.9)	90.1 (89.1–91.0)	28.6 (8.4–58.1)	96.0 (93.3–97.9)	23.4 (10.2–45.0)	96.9 (95.8–97.8)
	Platelia-Ab	29.2 (17.0–44.1)	74.9 (69.5–79.8)	16.1 (10.6–23.8)	86.5 (84.0–88.6)	23.5 (10.7–41.2)	74.9 (69.5–79.8)	9.9 (5.5–17.2)	89.3 (87.3–91.1)	42.9 (17.7–71.1)	75.1 (70.0–79.7)	6.8 (3.8–12.2)	96.8 (95.1–98.0)
	Serion-IgM	6.7 (1.4–18.3)	96.8 (94.0–98.5)	25.6 (8.8–55.0)	86.2 (85.2–87.2)	0.0 (0.0–10.9)	96.8 (94.0–98.5)	0.0	89.4 (89.2–89.6)	23.1 (5.0–53.8)	97.1 (94.6–98.7)	25.5 (9.5–52.8)	96.7 (95.6–97.5)
	Serion-IgG	13.3 (5.1–26.8)	88.7 (84.4–92.1)	16.3 (7.9–30.5)	86.1 (84.5–87.5)	3.1 (0.1–16.2)	88.7 (84.4–92.1)	3.0 (0.4–18.1)	9.0 (88.2–89.7)	38.5 (13.9–68.4)	89.5 (85.6–92.7)	13.5 (6.8–25.1)	97.1 (95.7–98.1)
	Serion-IgA	4.4 (0.5–15.1)	96.1 (93.1–98.0)	15.9 (4.1–45.1)	85.9 (85.0–86.7)	3.1 (0.1–16.2)	96.1 (93.1–98.0)	8.3 (1.2–40.5)	89.7 (89.1–90.3)	7.7 (0.2–36.0)	96.2 (93.4–98.0)	7.9 (1.2–38.0)	96.1 (95.4–96.6)
	CAGTA	22.9 (12.0–37.3)	78.4 (73.2–82.9)	14.9 (9.1–23.5)	86.0 (83.9–87.9)	17.6 (6.8–34.5)	78.4 (73.2–82.9)	8.7 (4.3–16.9)	89.1 (87.3–90.6)	35.7 (12.8–64.9)	78.8 (73.9–83.0)	6.7 (3.3–13.0)	96.6 (95.1–97.7)
Both serum samples positive	BDG	54.3 (39.0–69.1)	65.6 (59.7–71.1)	20.6 (16.0–26.1)	89.7 (86.3–92.4)	51.5 (33.5–69.2)	65.6 (59.7–71.1)	14.9 (10.8–20.2)	92.0 (89.0–94.3)	61.5 (31.6–86.1)	63.8 (58.2–69.1)	6.9 (4.5–10.5)	97.4 (95.0–98.7)
	Platelia-Mn	14.9 (6.2–28.3)	98.6 (96.5–99.6)	63.9 (35.0–85.3)	87.6 (86.2–88.8)	9.1 (1.9–24.3)	98.6 (96.5–99.6)	42.9 (14.9–76.2)	90.5 (89.5–91.4)	28.6 (8.4–58.1)	97.8 (95.6–99.1)	36.6 (16.0–63.5)	96.9 (95.7–97.8)
	Serion-Mn	8.5 (2.4–20.4)	99.3 (97.5–99.9)	67.0 (27.6–91.5)	86.9 (85.8–87.8)	3.0 (0.1–15.8)	99.3 (97.5–99.9)	33.3 (4.5–84.3)	90.0 (89.4–90.5)	21.4 (4.7–50.8)	99.1 (97.3–99.8)	50.3 (18.3–82.0)	96.6 (95.6–97.4)
	Platelia-Ab	16.7 (7.5–30.2)	80.7 (75.7–85.2)	12.5 (6.8–21.9)	85.5 (83.7–87.2)	11.8 (3.3–27.5)	80.8 (75.7–85.2)	6.8 (2.7–15.8)	88.5 (87.1–89.81)	28.6 (8.4–58.1)	81.6 (76.9–85.7)	6.4 (2.8–13.8)	96.3 (94.9–97.3)
	Serion-IgM	4.4 (0.5–15.1)	97.9 (95.6–99.2)	26.1 (6.8–62.9)	86.2 (85.4–87.0)	0.0 (0.0–10.3)	97.9 (95.6–99.2)	0.0	89.3 (89.1–89.5)	14.3 (1.8–42.8)	98.1 (96.0–99.3)	25.2 (7.0–60.4)	96.3 (95.5–97.0)
	Serion-IgG	10.4 (3.5–22.7)	92.4 (88.7–95.2)	18.3 (8.2–36.1)	86.3 (85.0–87.4)	2.9 (0.1–15.3)	92.4 (88.7–95.2)	4.3 (0.6–24.6)	89.0 (88.3–89.6)	28.6 (8.4–58.1)	92.9 (89.5–95.4)	15.0 (6.6–30.6)	96.7 (95.5–97.6)
	Serion-IgA	4.2 (0.5–14.3)	96.9 (94.2–98.6)	18.0 (4.7–49.6)	86.0 (85.3–86.8)	2.9 (0.1–15.3)	96.9 (94.2–98.6)	10.0 (1.4–46.0)	89.5 (88.9–90.0)	7.7 (0.2–36.0)	96.2 (93.4–98.0)	8.1 (1.2–38.6)	96.0 (95.3–96.5)
	CAGTA	20.8 (10.5–35.0)	83.7 (78.9–87.8)	17.3 (10.2–27.8)	86.6 (84.7–88.3)	14.7 (5.0–31.1)	83.7 (78.9–87.8)	9.6 (4.3–19.9)	89.3 (87.7–90.6)	35.7 (12.8–64.9)	83.9 (79.4–87.7)	8.6 (4.3–16.6)	96.8 (95.4–97.8)

^*a*^ BDG, β-(1→3)-D-glucan; Mn, mannan; Ab, antibody; CAGTA, *Candida albicans* germ tube antibodies; CI, confidence interval; Ppv, positive predictive value; Npv, negative predictive value; w/o, without.

At this point, it should be noted that owing to the limited number of candidemia cases, the 95% confidence intervals, especially for sensitivity, were wide; therefore, comparisons between biomarkers should be interpreted with caution.

As expected, the sensitivities of all biomarker assays were consistently higher for positivity criterion 1 than for positivity criterion 2, while the specificities showed the opposite behavior. The highest sensitivity for both criteria had the BDG assay (54.3%–71.4%). The assays for all other biomarkers had poor sensitivities, with values between 4.2% and 35.4% in ICI and 7.7% and 50.0% in candidemia. The sensitivities of all biomarkers were consistently higher for the diagnosis of candidemia than for the diagnosis of ICI, while the specificities were approximately the same for both entities. The Serion-IgA assay had the lowest sensitivity for all conditions (4.2%–7.7%). Specificities above 95% were achieved for positivity criterion 1 by Serion-Mn, Serion-IgM, and Serion-IgA, and for positivity criterion 2 additionally by Platelia-Mn. The BDG assay had the lowest specificity under all conditions (51.9%–65.6%). The high specificities at the manufacturer’s cutoff values also result in the highest PPV across all prevalences for Serion-Mn, Platelia-Mn, and Serion-IgM (Table S3).

### Diagnostic performance of single biomarkers at optimized cutoff values

The optimized cutoff values calculated by ROC analysis of the results of the first serum sample (Table 2) were mostly lower than the manufacturer’s cutoff values, except for BDG, where they were more than 3–4 times higher. The highest AUROC in ICI and candidemia had the antigen assays (BDG, Platelia-Mn, and Serion-Mn). Comparison of the AUROC of the three antigen assays showed that they were not statistically different (Table 3). However, the AUROC of Platelia-Mn and, to a lesser extent, BDG was significantly higher than that of some of the antibody tests (Serion-IgM, Serion-IgA, and CAGTA). Similarly, the AUROC of the antibody assays was not significantly different. Only Platelia-Ab in patients with candidemia showed a trend toward a higher AUROC compared to some of the other antibody assays (Serion-IgG and Serion-IgA). In terms of AUROC, Platelia-Mn was the superior biomarker, followed by BDG, Serion-Mn, and Platelia-Ab. Bootstrap-based analysis of cutoff optimization (highest Youden index) for Platelia-Mn and BDG is shown in Fig. S2.

**TABLE 2:** Optimized cutoff values with corresponding diagnostic performance for the measurement of the first serum sample^*a*^

Biomarker	Manufacturer’s cutoff value	Invasive *Candida* infection				Candidemia			
		Optimized cutoff value	Sensitivity (%) (95% CI)	Specificity (%) (95% CI)	AUROC (%) (95% CI) [95% bsCI]	Optimized cutoff value	Sensitivity (%) (95% CI)	Specificity (%) (95% CI)	AUROC (%) (95% CI) [95% bsCI]
BDG (pg/mL)	Negative < 60 Positive ≥ 80	>277	45.8 (31.4–60.8)	80.4 (75.4–84.8)	0.633 (0.579–0.685) [0.536–0.718]	>356	64.3 (35.1–87.2)	83.7 (79.2–87.5)	0.732 (0.682–0.779) [0.527–0.855]
Platelia-Mn (pg/mL)	Negative < 62.5 Positive ≥ 125	>85	35.4 (22.2–50.5)	90.7 (86.8–93.8)	0.646 (0.592–0.697) [0.545–0.728]	>108	64.3 (35.1–87.2)	90.8 (87.1–93.7)	0.779 (0.731–0.822) [0.599–0.880]
Serion-Mn (U/mL)	Negative < 1.4 Positive > 2.6	>1	25.0 (13.6–39.6)	93.8 (90.4–96.3)	0.602 (0.547–0.654) [0.500–0.696]	>0.7	50.0 (23.0–77.0)	79.7 (74.9–83.9)	0.668 (0.615–0.718) [0.461–0.789]
Platelia-Ab (AU/mL)	Negative < 5 Positive ≥ 10	>0.8	97.9 (88.9–99.9)	14.4 (10.6–19.0)	0.532 (0.477–0.586) [0.444–0.617]	>18.5	42.9 (17.7–71.1)	89.5 (85.7–92.6)	0.632 (0.578–0.684) [0.446–0.770]
Serion-IgM (U/mL)	Negative < 60 Positive > 80	>9	46.8 (32.1–61.9)	63.6 (57.8–69.1)	0.543 (0.489–0.597) [0.452–0.628]	>42	21.4 (4.7–50.8)	92.6 (89.2–95.2)	0.531 (0.476–0.585) [0.379–0.699]
Serion-IgG (U/mL)	Negative < 40 Positive > 100	≤31	61.7 (46.4–75.5)	29.9 (24.7–35.5)	0.507 (0.452–0.561) [0.416–0.605]	>119	35.7 (12.8–64.9)	93.5 (90.3–95.9)	0.597 (0.543–0.650) [0.409–0.753]
Serion-IgA (U/mL)	Negative < 60 Positive > 80	>6	53.2 (38.1–67.9)	58.1 (52.2–63.8)	0.545 (0.491–0.599) [0.454–0.627]	>1	85.7 (57.2–98.2)	30.3 (25.3–35.6)	0.547 (0.492–0.601) [0.390–0.691]
CAGTA (Index)	Negative < 0.9 Positive > 1.1	≤0.4	66.7 (51.6–79.6)	39.9 (34.2–45.7)	0.506 (0.451–0.560) [0.422–0.600]	>2.5	28.6 (8.4–58.1)	94.5 (91.4–96.7)	0.594 (0.540–0.647) [0.403–0.725]

^*a*^ The optimized cutoff is defined by the highest Youden index of the receiver operating characteristic (ROC) curve. BDG, β-(1→3)-D-glucan; Mn, mannan; U, units; Ab, antibody; AU, arbitrary units; CAGTA, *Candida albicans* germ tube antibodies; CI, confidence interval; AUROC, area under the ROC curve; bsCI, bootstrap confidence interval.

**TABLE 3:** Comparison of the area under the ROC curve (AUROC) of the biomarker results from the first serum sample^*a*^

Biomarker	BDG	Platelia-Mn	Serion-Mn	Platelia-Ab	Serion-IgM	Serion-IgG	Serion-IgA	CAGTA
BDG	---	0.803	0.589	*0.076*	0.154	*0.063*	0.147	**0.047**
Platelia-Mn	0.649	---	0.401	*0.051*	0.122	*0.058*	0.105	*0.052*
Serion-Mn	0.460	0.154	---	0.313	0.383	0.166	0.396	0.172
Platelia-Ab	0.446	0.131	0.741	---	0.739	0.802	0.647	0.790
Serion-IgM	0.108	**0.007**	0.189	0.121	---	0.661	0.962	0.649
Serion-IgG	0.300	*0.059*	0.518	*0.073*	0.290	---	0.653	0.972
Serion-IgA	0.114	**0.019**	0.250	*0.058*	0.783	0.270	---	0.644
CAGTA	0.277	*0.051*	0.491	0.122	0.361	0.881	0.261	---

^*a*^ *P*-values for the comparison of the AUROC of two biomarkers. *P*-values for diagnosis of ICI are shown right of the diagonal and for candidemia left of the diagonal. Statistically significant differences are printed in bold, and *P*-values between 0.05 and 0.08 are printed in italics. AUROC, area under the ROC curve; ICI, invasive *Candida* infection; BDG, β-(1→3)-D-glucan; Mn, mannan; Ab, antibody; CAGTA, *Candida albicans* germ tube antibodies.

Regardless of the cutoff values used, the sensitivities and specificities of the biomarkers were very different, which made direct comparisons difficult. Therefore, we calculated the performance for all biomarkers at a sensitivity of ~60% and a specificity of ~80% (Table 4). At a sensitivity of 60%, Platelia-Mn had the highest specificity in ICI (58.8%, 95% CI: 52.9–64.5, cutoff value 25 pg/mL) and candidemia (91.1%, 95% CI: 87.4–93.9, cutoff value 113 pg/mL). BDG also achieved a very good specificity of 88.9% (95% CI: 85.0–92.1) in candidemia (cutoff value 521 pg/mL). At a specificity of 80%, BDG and Platelia-Mn had the highest sensitivity in candidemia (64.3% in both cases, 95% CI: 35.1–87.2), while in ICI, BDG was superior to the other biomarkers (sensitivity 45.8%, 95% CI: 31.4–60.8, cutoff value 277 pg/mL).

**TABLE 4:** Diagnostic performance of the measurement of the first serum sample at specific values for sensitivity and specificity^*a*^

	Biomarker	Manufacturer’s cutoff value	Invasive *Candida* infection			Candidemia		
			Associated cutoff value	Sensitivity (%) (95% CI)	Specificity (%) (95% CI)	Associated cutoff value	Sensitivity (%) (95% CI)	Specificity (%) (95% CI)
Sensitivity of approximately 60%	BDG (pg/mL)	Negative < 60 Positive ≥ 80	>70	60.4 (45.3–74.2)	52.6 (46.7–58.4)	>521	57.1 (28.9–82.3)	88.9 (85.0–92.1)
	Platelia-Mn (pg/mL)	Negative < 62.5 Positive ≥ 125	>25	60.4 (45.3–74.2)	58.8 (52.9–64.5)	>113	57.1 (28.9–82.3)	91.1 (87.4–93.9)
	Serion-Mn (U/mL)	Negative < 1.4 Positive > 2.6	>0.5	66.7 (51.6–79.6)	49.8 (43.9–55.7)	>0.6	50.0 (23.0–77.0)	68.6 (63.3–73.6)
	Platelia-Ab (AU/mL)	Negative < 5 Positive ≥ 10	>4	60.4 (45.3–74.2)	46.7 (40.9–52.6)	>5.4	57.1 (28.9–82.3)	53.5 (48.0–59.1)
	Serion-IgM (U/mL)	Negative < 60 Positive > 80	>4	66.0 (50.7–79.1)	42.3 (36.5–48.2)	>4	57.1 (28.9–82.3)	41.1 (35.6–46.6)
	Serion-IgG (U/mL)	Negative < 40 Positive > 100	≤31	61.7 (46.4–75.5)	29.9 (24.7–35.5)	>17	57.1 (28.9–82.3)	54.0 (48.4–59.5)
	Serion-IgA (U/mL)	Negative < 60 Positive > 80	>3	59.6 (44.3–73.6)	43.3 (37.5–49.2)	>2	57.1 (28.9–82.3)	36.7 (31.5–42.2)
	CAGTA (Index)	Negative < 0.9 Positive > 1.1	≤0.4	66.7 (51.6–79.6)	39.9 (34.2–45.7)	>0.2	57.1 (28.9–82.3)	48.6 (43.1–54.2)
Specificity of approximately 80%	BDG (pg/mL)	Negative < 60 Positive ≥ 80	>277	45.8 (31.4–60.8)	80.4 (75.4–84.8)	>286	64.3 (35.1–87.2)	80.0 (75.2–84.2)
	Platelia-Mn (pg/mL)	Negative < 62.5 Positive ≥ 125	>47	37.5 (24.0–52.6)	80.1 (75.0–84.5)	>48	64.3 (35.1–87.2)	80.3 (75.6–84.5)
	Serion-Mn (U/mL)	Negative < 1.4 Positive > 2.6	>0.7	37.5 (24.0–52.6)	81.1 (76.1–85.4)	>0.7	50.0 (23.0–77.0)	79.7 (74.9–83.9)
	Platelia-Ab (AU/mL)	Negative < 5 Positive ≥ 10	>11.1	27.1 (15.3–41.8)	80.1 (75.0–84.5)	>11.1	42.9 (17.7–71.1)	80.0 (75.2–84.2)
	Serion-IgM (U/mL)	Negative < 60 Positive > 80	>19	23.4 (12.3–38.0)	80.4 (75.4–84.8)	>19	28.6 (8.4–58.1)	80.3 (75.5–84.4)
	Serion-IgG (U/mL)	Negative < 40 Positive > 100	≤3	25.5 (13.9–40.3)	81.4 (76.5–85.7)	>47	35.7 (12.8–64.9)	79.9 (75.2–84.2)
	Serion-IgA (U/mL)	Negative < 60 Positive > 80	>19	23.4 (12.3–38.0)	80.1 (75.0–84.5)	>20	14.3 (1.8–42.8)	80.9 (76.2–85.0)
	CAGTA (Index)	Negative < 0.9 Positive > 1.1	≤0	25.0 (13.6–39.6)	74.6 (69.2–79.5)	>1.1	35.7 (12.8–64.9)	79.7 (74.9–83.9)

^*a*^ BDG, β-(1→3)-D-glucan; Mn, mannan; U, units; Ab, antibody; AU, arbitrary units; CAGTA, *Candida albicans* germ tube antibodies; CI, confidence interval.

Due to the higher cutoff value for BDG and the associated increase in specificity, analysis of the PPV across different prevalences showed that all antigen assays now consistently exhibited the highest PPV (Table S3).

The diagnostic performance of the biomarkers in patients with and without *Candida* colonization, as well as in patients with and without recent abdominal surgery, is shown in Tables S4 and S5. Antigen assays showed higher sensitivity in patients with *Candida* colonization, although differences were not statistically significant (BDG: *P* = 0.137; Platelia-Mn: *P* = 0.075; and Serion-Mn: *P* = 0.279), while specificity was unaffected. In terms of colonization status, the antibody tests showed inconsistent results. In patients who had undergone recent abdominal surgery (<7 days), antigen sensitivity was also higher, reaching statistical significance only for Platelia-Mn (BDG: *P* = 0.361; Platelia-Mn: *P* = 0.045; and Serion-Mn: *P* =0.071).

### Diagnostic performance of biomarker combinations

For patients with ICI, multiple binary logistic regression analyses showed that when the first serum was tested simultaneously with several biomarkers, only the combination of BDG and Platelia-Mn made a significant contribution to the prediction model. The AUROC of the BDG and Platelia-Mn combination was 0.648 (95% CI: 0.558–0.739), with optimism-corrected AUROC after bootstrapping of 0.643 (95% bootstrap CI: 0.548–0.733) and a Brier Score of 0.122 (95% CI: 0.086–0.145). However, this AUROC was not significantly different (*P* = 0.942) from that of Platelia-Mn testing alone (AUROC: 0.646, 95% CI: 0.592–0.697; AUROC with bootstrapping: 0.645, 95% CI: 0.557–0.740; Brier Score 0.122, 95% CI: 0.086–0.144). Optimism after internal validation was equal to 0.008. For patients with candidemia, multiple binary logistic regression analyses showed that BDG and Serion-IgG were the optimal combination for simultaneous testing. The AUROC in this case was 0.761 (95% CI: 0.620–0.901), with an optimism-corrected AUROC after bootstrapping of 0.738 (95% bootstrap CI: 0.533–0.883) and a Brier Score of 0.040 (95% CI: 0.021–0.057). However, this AUROC was not significantly different (*P* = 0.523) from that of BDG testing alone (AUROC: 0.732, 95% CI: 0.682–0.779; AUROC with bootstrapping: 0.736, 95% CI: 0.544–0.885; Brier Score: 0.041, 95% CI: 0.022–0.061).

**Fig 4:**
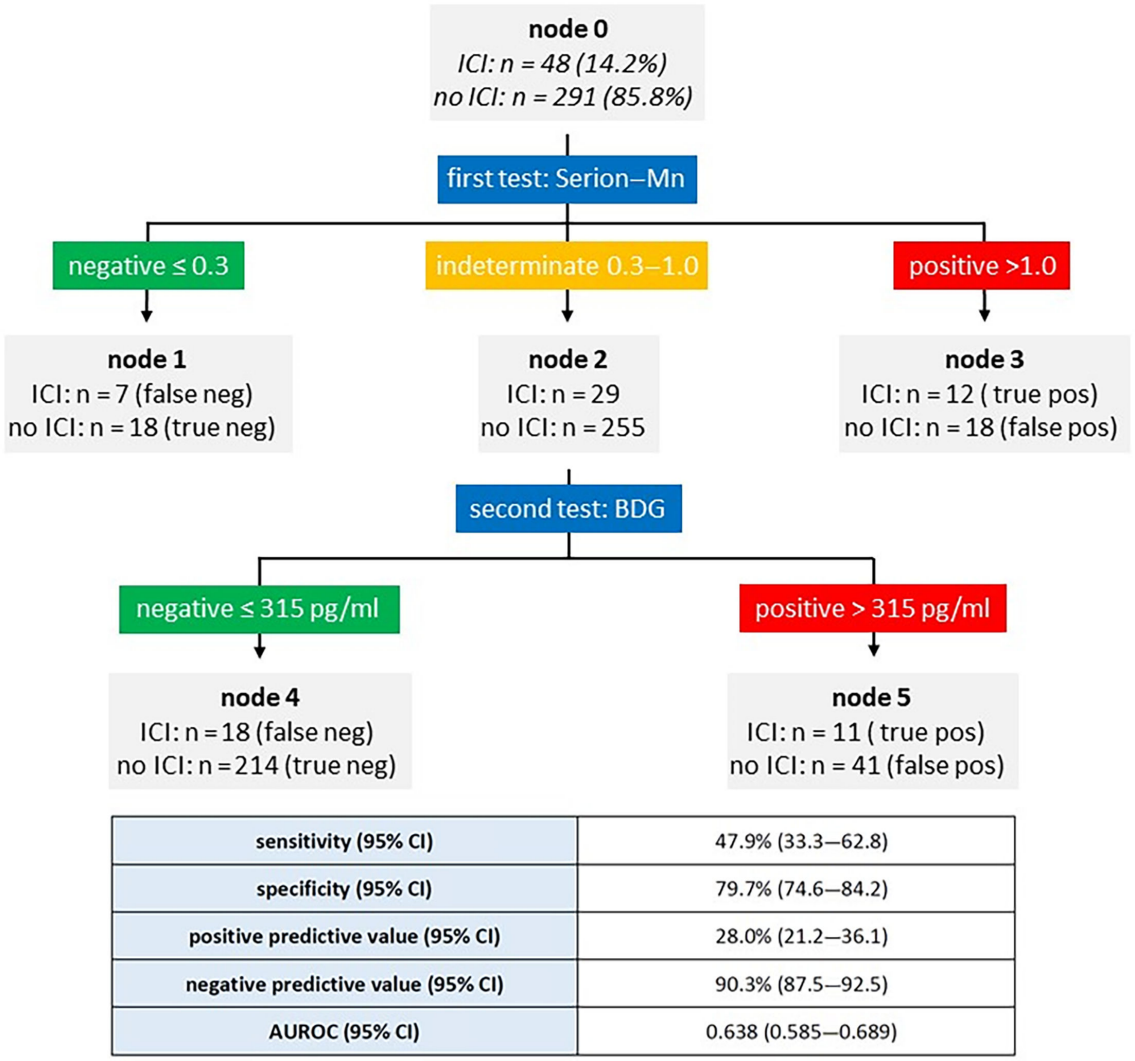
Decision tree for the diagnosis of ICI from the first serum sample. Node 0 represents the whole study population. Testing with Serion-Mn leads to nodes 1, 2, and 3. The sera of node 2 are then tested with BDG. The final result includes the patients from nodes 1, 3, 4, and 5. The diagnostic performance is shown at the bottom. ICI, invasive *Candida* infection; CI, confidence interval; AUROC, area under the ROC curve.

The optimal sequence of two consecutive biomarker tests of the first serum was analyzed using supervised machine learning, generating a decision tree. For the diagnosis of ICI, the best course of action was to use the Serion-Mn assay and then retest those sera with the BDG assay, which had a Serion-Mn value between 0.3 and 1.0 pg/mL (Fig. 4). This approach resulted in a sensitivity of 47.9% (95% CI: 33.3–62.8), a specificity of 79.7% (95% CI: 74.6–84.2), and an AUROC of 0.638 (0.585–0.689). For the diagnosis of candidemia, a single BDG measurement with a cutoff value of >315 pg/mL was superior to consecutive testing (sensitivity 64.3% [95% CI: 35.1–87.2], specificity 82.2% [95% CI: 77.5–86.2]). Again, the AUROC of combination testing was lower than that of Platelia-Mn testing alone.

The diagnostic performance of the simultaneous combination of two assays at the manufacturer’s cutoff values (one positive assay from one serum is sufficient for positivity) is shown in Table S6 and Venn diagrams in Fig. S1.

### Decision curve analysis

Decision curve plots for the diagnosis of ICI (BDG, Platelia-Mn, and the combination of both) and candidemia (BDG and Serion-IgG) are shown in Fig. S3. For ICI, BDG, Platelia-Mn, and their combination exhibit a nearly identical net benefit, which remains very low across a threshold probability range of approximately 13%–30%. For candidemia, both BDG and Serion-IgG show very low net benefit between threshold probabilities of approximately 3%–10%. Above a threshold probability of 10%, BDG demonstrates a negative net benefit.

## DISCUSSION

We analyzed sera from the CandiSep trial with assays for BDG, Mn, and anti-*Candida*-Ab to evaluate their diagnostic utility for ICI. We assessed single biomarker performance and combinations of two biomarkers, both simultaneously and sequentially.

Sensitivity for diagnosing candidemia was consistently higher than for deep-seated candidiasis, with nearly similar specificity. In addition, specificity increased when both serum samples tested positive, confirming previous reports (26–28).

Only the fungal antigen tests, but not the anti-*Candida*-Ab tests, yielded significantly higher values for patients with ICI and candidemia compared to patients without fungal infection. Furthermore, anti-*Candida* Ab levels in colonized patients without infection were similar to those in patients with ICI, and both were higher than in non-infected patients without colonization. This observation suggests that the colonization status, rather than the presence of ICI, determines the level of anti-*Candida*-Ab. Previous studies investigating the influence of colonization on antigen and antibody levels have produced conflicting results. Poissy *et al*. and Mokaddas *et al*. could not find a significant influence of colonization on BDG and mannan levels in adult patients (12, 29). Additionally, neonates with and without rectal *Candida* colonization had similar Platelia-Mn levels (30). With regard to anti-*Candida*-Ab, Mattsby-Baltzer *et al*. found that IgG1 and IgG2 antibodies against *Candida* cell wall fragments and mannan had similar serum levels in patients with ICI and in patients with heavy *Candida* colonization, whereas non-colonized patients had much lower levels (31). Standaert-Vitse *et al*. also showed that the concentration of anti-mannan antibodies in serum correlated with *Candida* colonization in patients with Crohn’s disease and their healthy relatives (32). In contrast, Leon *et al*. found that the median BDG but not CAGTA level was higher in colonized than in non-colonized patients (33). Interestingly, this changed when the maximum BDG values were analyzed. Then, the maximum BDG values between colonized and non-colonized patients were comparable, and both were much lower than in ICI patients. In a subsequent study, Leon *et al*. distinguished between low-grade and high-grade colonization and found that the proportion of patients with positive antigen and antibody tests was significantly higher in cases of high-grade colonization. For Platelia-Mn and CAGTA, the positivity rate of high-grade colonized patients was even higher than in patients with intra-abdominal candidiasis (28). Both studies examined patients with severe abdominal conditions, and it must be taken into account that intestinal surgery alone can lead to increased Platelia-Ab counts (34). In our opinion, elevated anti-*Candida*-Ab levels might only serve as an indirect indicator of ICI because *Candida* colonization leads to elevated anti-*Candida*-Ab levels and is itself a major risk factor for ICI. In summary, our data show that anti-*Candida*-Ab assays alone are only of limited use for the diagnosis of ICI in our setting.

If only a single biomarker is used at the manufacturer’s cutoff values, BDG is the one not only with the highest sensitivity but also with an unacceptably low specificity. This restriction of the BDG assay was the reason that in the CandiSep trial, almost 50% of patients in the BDG-guided group received antifungal therapy, although only 14% had an ICI. BDG-guided therapy caused overtreatment without a benefit in mortality (19). The high number of false-positive BDG results could be due, at least in part, to the fact that many surgical ICUs participated in the CandiSep trial, which means that the majority of patients had recently undergone surgery (69.6%). It is known, however, that surgical materials such as sponges and gauze release BDG into the environment, which is why most patients have elevated BDG levels for at least 3–7 days after major surgery (35, 36). In addition, many patients with septic shock in Germany receive albumin, which can be another reason for false-positive BDG levels (37). Both factors could be partly responsible for the low BDG specificity. What contradicts this assumption is that our analysis did not reveal a significant difference in BDG specificity between patients who had undergone abdominal surgery and those who had not.

Although the BDG value at the manufacturer’s cutoff value appears unsuitable for initiating antifungal therapy, BDG can still be used to discontinue it in the event of a negative result. This approach successfully reduced antifungal consumption in two randomized studies in ICU patients (38, 39) but failed in another randomized study conducted by our group, even leading to a temporary interruption of antifungal therapy in one patient with candidemia (40). In contrast to BDG, all other biomarkers in our study showed poor sensitivities but very good to excellent specificities, which indicates that the manufacturer’s cutoff values are inadequate. In the case of BDG, it seems logical that a single cutoff value may not be optimal for the diagnosis of aspergillosis, ICI, or *Pneumocystis jirovecii* pneumonia, three fungal diseases that have completely different mean BDG values. To improve the cutoff values, we carried out an ROC analysis, and indeed, all the optimized cutoff values were significantly lower than those of the manufacturers, except for BDG. The results of the CandiSep trial suggested that the specificity of a biomarker should be at least 80% to prevent overtreatment. A cutoff value of ≥280 pg/mL for BDG, ≥50 pg/mL for Platelia-Mn, and >0.7 U/mL for Serion-Mn resulted in such specificity and showed sensitivities between 38% and 46% in patients with ICI and 50% and 64% in patients with candidemia. The anti-*Candida* Ab assays were again inferior and achieved sensitivities of only 23%–27% in ICI and 14%–43% in candidemia. Similarly, the AUROC showed no significant difference between the antigen assays, but in some cases, between the antigen assays and the anti-*Candida*-Ab assays.

To our knowledge, there are seven previous prospective studies that have investigated the performance of biomarkers in ICU patients with ICI. An overview of those studies and their results is provided in Table S7. Four of them studied only BDG (41–44), two BDG and CAGTA (14, 33), and one BDG, Platelia-Mn, Platelia-Ab, and CAGTA (28). Two of the studies evaluating more than one biomarker were conducted by Leon *et al*. in 2012 and 2016 in ICU patients with severe abdominal conditions, with serum samples collected twice weekly. In the study from 2012, they used a BDG cutoff value of 260 pg/mL and found a sensitivity of 51.6% and a specificity of 86.9%. These results are quite similar to our results using the optimized BDG cutoff value of 277 pg/mL (sensitivity 45.8%; specificity 80.4%). They also tested their sera for CAGTA with an immunofluorescence assay (IFA), which showed a sensitivity and specificity of 71.0% and 57.3%, respectively. In our study, the CAGTA-Monotest had a similar sensitivity of 66.7%, but a much lower specificity of 39.9%, an observation that was also reported by Parra-Sanchez *et al*. in their direct comparison of the CAGTA-IFA and the CAGTA-Monotest (27). In the study by Leon *et al*. from 2016, the Platelia-Mn sensitivity of 43.3% was slightly higher, and the specificity of 67.3% slightly lower than the respective values in our work. This difference is explained by the use of a lower cutoff value of 60 pg/mL by Leon *et al*., which is remarkably similar to the optimized cutoff value of 50 pg/mL defined by us. Finally, the sensitivity of the Platelia-Ab assay was low in the study from 2016 (25.8%), which is consistent with our results (29.2%).

As another important result of our study, we noted that neither the simultaneous nor the sequential testing of two biomarkers resulted in an AUROC that was significantly different from the diagnostic performance of single biomarker measurements. Consequently, our data suggest that combination testing is not helpful. Interestingly, Leon *et al*. also performed a decision tree analysis in 2012 and found that initial testing with BDG at a cutoff value of ≥260 pg/mL, and subsequent testing of BDG-negative sera with CAGTA-IFT resulted in a sensitivity of 90.3% and a specificity of 54.8%, which was superior to the analysis of the individual biomarkers. These discrepancies show that the results of decision tree analyses may only be optimal for the specific patient population studied and that they should be considered exploratory until they are confirmed by independent sample sets.

Our study also has some limitations. First, the number of ICI and candidemia cases is limited, which leads to a wide 95% CI, especially for the sensitivity in candidemia. Minor differences in the diagnostic performance of two biomarkers could therefore also be random fluctuations, and the fact that no significant differences between two biomarkers were found could change when larger case groups are examined. Second, the CandiSep trial examined a very well-defined population, that is, critically ill patients with severe sepsis or septic shock and an increased risk of ICI. Therefore, our considerations and conclusions apply only to a limited extent to other patient populations or specific ICU subpopulations. Third, BDG was measured during the CandiSep trial (09/2016 to 09/2019), whereas the other biomarkers were analyzed in batch between 02/2019 and 12/2019. In the worst-case scenario, this resulted in a time gap of up to 3 years between the BDG measurements and the assessment of the other biomarkers. During this period, degradation could have reduced the concentrations of Platelia-Mn and Serion-Mn. A detailed discussion on the likelihood of degradation, based on a small test series of sera stored for 5 years at –20°C, is provided in the supplemental material. Fourth, in the CandiSep trial, ICI diagnosis was only assessed up to 96 h after inclusion. Consequently, ICI cases diagnosed beyond this time window may have been missed, potentially leading to an underestimation of specificity.

In summary, in our cohort of ICU patients, only the fungal antigen assays yielded significantly higher biomarker values for patients with ICI and candidemia compared to uninfected controls. Anti-*Candida* Ab levels may be induced by *Candida* colonization and appear to be of limited utility for the diagnosis of ICI. The manufacturer’s cutoff values may not be optimal for diagnosing ICI or candidemia and could require adjustment for all biomarkers except BDG, for which a higher cutoff might be appropriate. Based on our data, a specificity of 80% would be achieved by a cutoff value of ≥280 pg/mL for BDG, ≥50 pg/mL for Platelia-Mn, and >0.7 U/mL for Serion-Mn. The antigen tests showed comparable AUROC values, with small potential advantages for BDG and Platelia-Mn. Simultaneous or sequential testing of two different biomarkers does not appear to be superior to single biomarker testing and therefore may not be necessary. Due to the limited number of ICI and candidemia cases, our results should be confirmed in a larger study.

## Data Availability

The original data sets of this study are available from the corresponding author (J.H.) upon reasonable request.

## SUPPLEMENTAL MATERIAL

Supplemental material jcm.01500-25-s0001.docx Tables S1 to S7; Fig. S1 to S3.

ASM does not own the copyrights to Supplemental Material that may be linked to, or accessed through, an article. The authors have granted ASM a non-exclusive, world-wide license to publish the Supplemental Material files. Please contact the corresponding author directly for reuse.

## References

[1] Koehler P, Stecher M, Cornely OA, Koehler D, Vehreschild M, Bohlius J, Wisplinghoff H, Vehreschild JJ. 2019. Morbidity and mortality of candidaemia in Europe: an epidemiologic meta-analysis. Clin Microbiol Infect 25:1200–1212. doi:10.1016/j.cmi.2019.04.024 31039444

[2] Gouel-Cheron A, Swihart BJ, Warner S, Mathew L, Strich JR, Mancera A, Follmann D, Kadri SS. 2022. Epidemiology of ICU-onset bloodstream infection: prevalence, pathogens, and risk factors among 150,948 ICU patients at 85 U.S. hospitals. Crit Care Med 50:1725–1736. doi:10.1097/CCM.0000000000005662 36190259 PMC10829879

[3] Träger J, Dräger S, Mihai S, Cipa F, Busse Grawitz A, Epting T, Meyer R, Rappold E, Held J. 2023. Detailed β-(1→3)-D-glucan and mannan antigen kinetics in patients with candidemia. J Clin Microbiol 61:e0059823. doi:10.1128/jcm.00598-23 37823667 PMC10662340

[4] Morrell M, Fraser VJ, Kollef MH. 2005. Delaying the empiric treatment of Candida bloodstream infection until positive blood culture results are obtained: a potential risk factor for hospital mortality. Antimicrob Agents Chemother 49:3640–3645. doi:10.1128/AAC.49.9.3640-3645.2005 16127033 PMC1195428

[5] Clancy CJ, Nguyen MH. 2018. Non-culture diagnostics for invasive candidiasis: promise and unintended consequences. J Fungi (Basel) 4:27. doi:10.3390/jof4010027 29463043 PMC5872330

[6] Mikulska M, Calandra T, Sanguinetti M, Poulain D, Viscoli C, Leukemia G. 2010. The use of mannan antigen and anti-mannan antibodies in the diagnosis of invasive candidiasis: recommendations from the Third European Conference on Infections in Leukemia. Crit Care 14:R222. doi:10.1186/cc9365 21143834 PMC3219989

[7] Finkelman MA. 2020. Specificity influences in (1→3)-β-d-glucan-supported diagnosis of invasive fungal disease. J Fungi (Basel) 7:14. doi:10.3390/jof7010014 33383818 PMC7824349

[8] Kondori N, Edebo L, Mattsby-Baltzer I. 2004. Circulating β (1-3) glucan and immunoglobulin G subclass antibodies to Candida albicans cell wall antigens in patients with systemic candidiasis. Clin Diagn Lab Immunol 11:344–350. doi:10.1128/cdli.11.2.344-350.2004 15013986 PMC371202

[9] Alam FF, Mustafa AS, Khan ZU. 2007. Comparative evaluation of (1, 3)-β-D-glucan, mannan and anti-mannan antibodies, and Candida species-specific snPCR in patients with candidemia. BMC Infect Dis 7:103. doi:10.1186/1471-2334-7-103 17784947 PMC2075513

[10] Martínez-Jiménez MC, Muñoz P, Valerio M, Alonso R, Martos C, Guinea J, Bouza E. 2015. Candida biomarkers in patients with candidaemia and bacteraemia. J Antimicrob Chemother 70:2354–2361. doi:10.1093/jac/dkv090 25900160

[11] Eades CP, Bakri A, Lau JCY, Moore CB, Novak-Frazer L, Richardson MD, Rautemaa-Richardson R. 2023. Comparison of β-1-3-D-glucan and Candida mannan biomarker assays with serological tests for the diagnosis of candidemia. J Fungi (Basel) 9:813. doi:10.3390/jof9080813 37623584 PMC10455369

[12] Mokaddas E, Khan ZU, Ahmad S, Nampoory MRN, Burhamah M. 2011. Value of (1-3)-β-D-glucan, Candida mannan and Candida DNA detection in the diagnosis of candidaemia. Clin Microbiol Infect 17:1549–1553. doi:10.1111/j.1469-0691.2011.03608.x 21883664

[13] Montagna MT, Coretti C, Lovero G, De Giglio O, Montagna O, Laforgia N, Santoro N, Caggiano G. 2011. Diagnostic performance of 1→3-β-D-glucan in neonatal and pediatric patients with candidemia. Int J Mol Sci 12:5871–5877. doi:10.3390/ijms12095871 22016633 PMC3189757

[14] Martín-Mazuelos E, Loza A, Castro C, Macías D, Zakariya I, Saavedra P, Ruiz-Santana S, Marín E, León C. 2015. β-d-Glucan and Candida albicans germ tube antibody in ICU patients with invasive candidiasis. Intensive Care Med 41:1424–1432. doi:10.1007/s00134-015-3922-y 26134359

[15] Cornely OA, Sprute R, Bassetti M, Chen SC-A, Groll AH, Kurzai O, Lass-Flörl C, Ostrosky-Zeichner L, Rautemaa-Richardson R, Revathi G, et al.. 2025. Global guideline for the diagnosis and management of candidiasis: an initiative of the ECMM in cooperation with ISHAM and ASM. Lancet Infect Dis 25:e280–e293. doi:10.1016/S1473-3099(24)00749-7 39956121

[16] Bassetti M, Garnacho-Montero J, Calandra T, Kullberg B, Dimopoulos G, Azoulay E, Chakrabarti A, Kett D, Leon C, Ostrosky-Zeichner L, Sanguinetti M, Timsit JF, Richardson MD, Shorr A, Cornely OA. 2017. Intensive care medicine research agenda on invasive fungal infection in critically ill patients. Intensive Care Med 43:1225–1238. doi:10.1007/s00134-017-4731-2 28255613

[17] Montravers P, Tashk P, Tran Dinh A. 2017. Unmet needs in the management of intra-abdominal infections. Expert Rev Anti Infect Ther 15:839–850. doi:10.1080/14787210.2017.1372750 28841096

[18] Bloos F, Held J, Schlattmann P, Brillinger N, Kurzai O, Cornely OA, Thomas-Rüddel D. 2018. (1,3)-β-D-glucan-based diagnosis of invasive Candida infection versus culture-based diagnosis in patients with sepsis and with an increased risk of invasive Candida infection (CandiSep): study protocol for a randomized controlled trial. Trials 19:472. doi:10.1186/s13063-018-2868-0 30180873 PMC6124015

[19] Bloos F, Held J, Kluge S, Simon P, Kogelmann K, de Heer G, Kuhn S-O, Jarczak D, Motsch J, Hempel G, et al.. 2022. (1 → 3)-β-d-Glucan-guided antifungal therapy in adults with sepsis: the CandiSep randomized clinical trial. Intensive Care Med 48:865–875. doi:10.1007/s00134-022-06733-x 35708758 PMC9273538

[20] Vickers AJ, Elkin EB. 2006. Decision curve analysis: a novel method for evaluating prediction models. Med Decis Making 26:565–574. doi:10.1177/0272989X06295361 17099194 PMC2577036

[21] Harrell FE, Lee KL, Mark DB. 1996. Multivariable prognostic models: issues in developing models, evaluating assumptions and adequacy, and measuring and reducing errors. Stat Med 15:361–387. doi:10.1002/(SICI)1097-0258(19960229)15:4<361::AID-SIM168>3.0.CO;2-4 8668867

[22] R Core Team. 2024. R: a language and environment for statistical computing_. R Foundation for Statistical Computing, Vienna, Austria. https://www.R-project.org/, https://CRAN.R-project.org/package=rms.

[23] Thiele C, Hirschfeld G. 2021. Cutpointr: improved estimation and validation of optimal cutpoints in R. J Stat Softw 98. doi:10.18637/jss.v098.i11

[24] Sjoberg D. 2025. dcurves: decision curve analysis for model evaluation_. R package version 0.5.1 hCR-popd

[25] Harrell Jr FE. 2025. rms: regression modeling strategies_. R package version 8.0-0. https://CRAN.R-project.org/package=rms.

[26] Nguyen MH, Wissel MC, Shields RK, Salomoni MA, Hao B, Press EG, Shields RM, Cheng S, Mitsani D, Vadnerkar A, Silveira FP, Kleiboeker SB, Clancy CJ. 2012. Performance of Candida real-time polymerase chain reaction, β-D-glucan assay, and blood cultures in the diagnosis of invasive candidiasis. Clin Infect Dis 54:1240–1248. doi:10.1093/cid/cis200 22431804

[27] Parra-Sánchez M, Zakariya-Yousef Breval I, Castro Méndez C, García-Rey S, Loza Vazquez A, Úbeda Iglesias A, Macías Guerrero D, Romero Mejías A, León Gil C, Martín-Mazuelos E, CAVA Trem Study Group. 2017. Candida albicans germ-tube antibody: evaluation of a new automatic assay for diagnosing invasive candidiasis in ICU patients. Mycopathologia 182:645–652. doi:10.1007/s11046-017-0125-9 28378240

[28] León C, Ruiz-Santana S, Saavedra P, Castro C, Loza A, Zakariya I, Úbeda A, Parra M, Macías D, Tomás JI, Rezusta A, Rodríguez A, Gómez F, Martín-Mazuelos E, Cava Trem Study Group. 2016. Contribution of Candida biomarkers and DNA detection for the diagnosis of invasive candidiasis in ICU patients with severe abdominal conditions. Crit Care 20:149. doi:10.1186/s13054-016-1324-3 27181045 PMC4867537

[29] Poissy J, Sendid B, Damiens S, Ichi Ishibashi K, François N, Kauv M, Favory R, Mathieu D, Poulain D. 2014. Presence of Candida cell wall derived polysaccharides in the sera of intensive care unit patients: relation with candidaemia and Candida colonisation. Crit Care 18:R135. doi:10.1186/cc13953 24975380 PMC4227034

[30] Bourika V, Siahanidou T, Theodoridou K, Tsakris A, Vrioni G, Michos A. 2024. Evaluation of the mannan antigen assay in neonates with or without Candida albicans colonization. Med Mycol 62:myad138. doi:10.1093/mmy/myad138 38167789 PMC10818226

[31] Mattsby-Baltzer I, Pinel C, Yugueros Marcos J, Kondori N, Potton L, Thiebaut-Bertrand A, Pelloux H, Cornet M. 2015. IgG1 anti-cell wall and IgG2 anti-phosphopeptidomannan antibodies in the diagnosis of invasive candidiasis and heavy Candida colonization. Med Mycol 53:725–735. doi:10.1093/mmy/myv037 26162476

[32] Standaert-Vitse A, Sendid B, Joossens M, François N, Vandewalle-El Khoury P, Branche J, Van Kruiningen H, Jouault T, Rutgeerts P, Gower-Rousseau C, Libersa C, Neut C, Broly F, Chamaillard M, Vermeire S, Poulain D, Colombel J-F. 2009. Candida albicans colonization and ASCA in familial Crohn’s disease. Am J Gastroenterol 104:1745–1753. doi:10.1038/ajg.2009.225 19471251

[33] León C, Ruiz-Santana S, Saavedra P, Castro C, Ubeda A, Loza A, Martín-Mazuelos E, Blanco A, Jerez V, Ballús J, Alvarez-Rocha L, Utande-Vázquez A, Fariñas O. 2012. Value of β-d-glucan and Candida albicans germ tube antibody for discriminating between Candida colonization and invasive candidiasis in patients with severe abdominal conditions. Intensive Care Med 38:1315–1325. doi:10.1007/s00134-012-2616-y 22752333

[34] Warnock DW, Speller DC, Finan PJ, Vellacott KD, Phillips MN. 1979. Antibodies to Candida species after operations on the large intestine: observations on the association with oral and faecal yeast colonization. Sabouraudia 17:405–414. doi:10.1080/00362177985380601 120986

[35] Szyszkowitz A, Zurl C, Herzeg A, Berger A, Gemes G, Mitteregger M, Prüller F, Prattes J, Zollner-Schwetz I, Valentin T, Hoenigl M, Krause R. 2018. Serum 1,3-beta-D-glucan values during and after laparoscopic and open intestinal surgery. Open Forum Infect Dis 5:ofy296. doi:10.1093/ofid/ofy296 30568978 PMC6290064

[36] Mohr JF, Sims C, Paetznick V, Rodriguez J, Finkelman MA, Rex JH, Ostrosky-Zeichner L. 2011. Prospective survey of (1→3)-β-d-glucan and its relationship to invasive candidiasis in the surgical intensive care unit setting. J Clin Microbiol 49:58–61. doi:10.1128/JCM.01240-10 21048005 PMC3020467

[37] Held J, Wagner D. 2011. β-d-Glucan kinetics for the assessment of treatment response in Pneumocystis jirovecii pneumonia. Clin Microbiol Infect 17:1118–1122. doi:10.1111/j.1469-0691.2010.03452.x 21446990

[38] Rouzé A, Loridant S, Poissy J, Dervaux B, Sendid B, Cornu M, Nseir S, S-TAFE study group. 2017. Biomarker-based strategy for early discontinuation of empirical antifungal treatment in critically ill patients: a randomized controlled trial. Intensive Care Med 43:1668–1677. doi:10.1007/s00134-017-4932-8 28936678

[39] De Pascale G, Posteraro B, D’Arrigo S, Spinazzola G, Gaspari R, Bello G, Montini LM, Cutuli SL, Grieco DL, Di Gravio V, De Angelis G, Torelli R, De Carolis E, Tumbarello M, Sanguinetti M, Antonelli M. 2020. (1,3)-β-D-Glucan-based empirical antifungal interruption in suspected invasive candidiasis: a randomized trial. Crit Care 24:550. doi:10.1186/s13054-020-03265-y 32891170 PMC7487510

[40] Erb T, Mihai S, Strauß R, Herbst L, Castellanos I, Diesch K, Cipa F, Bihlmaier K, Lang A-K, Ganslmayer M, Willam C, Bremer F, Fürst J, Beyer C, Bogdan C, Rath A, Held J. 2023. β-(1→3)-D-glucan- and mannan-guided early termination of antifungal therapy in ICU patients: a randomized controlled study. Antimicrob Agents Chemother 67:e0072523. doi:10.1128/aac.00725-23 37823695 PMC10648872

[41] Posteraro B, De Pascale G, Tumbarello M, Torelli R, Pennisi MA, Bello G, Maviglia R, Fadda G, Sanguinetti M, Antonelli M. 2011. Early diagnosis of candidemia in intensive care unit patients with sepsis: a prospective comparison of (1→3)-β-D-glucan assay, Candida score, and colonization index. Crit Care 15:R249. doi:10.1186/cc10507 22018278 PMC3334800

[42] Christner M, Abdennadher B, Wichmann D, Kluge S, Pepić A, Aepfelbacher M, Rohde H, Olearo F. 2024. The added value of (1,3)-β-D-glucan for the diagnosis of Invasive candidiasis in ICU patients: a prospective cohort study. Infection 52:73–81. doi:10.1007/s15010-023-02053-4 37322388 PMC10811116

[43] Tissot F, Lamoth F, Hauser PM, Orasch C, Flückiger U, Siegemund M, Zimmerli S, Calandra T, Bille J, Eggimann P, Marchetti O, Fungal Infection Network of Switzerland (FUNGINOS). 2013. β-glucan antigenemia anticipates diagnosis of blood culture-negative intraabdominal candidiasis. Am J Respir Crit Care Med 188:1100–1109. doi:10.1164/rccm.201211-2069OC 23782027

[44] Novy E, Rivière J, Nguyen M, Arfeuille G, Louis G, Bouhemad B, Pottecher J, Hecketsweiler S, Germain A, Laithier F-X, Losser M-R, Debourgogne A, Bernard Y, Rousseau H, Baumann C, Luc A, Birckener J, Machouart M-C, Guerci P. 2023. Combination of serum and peritoneal 1.3-beta-d-glucan can rule out intra-abdominal candidiasis in surgical critically ill patients: a multicenter prospective study. Crit Care 27:470. doi:10.1186/s13054-023-04761-7 38037130 PMC10691030

