# Supplementary material for: Diagnostic performance of β-(1→3)-D-glucan, two Candida antigen, and five anti-Candida antibody assays in ICU patients with sepsis and high risk for invasive candidiasis: a secondary endpoint of the CandiSep randomized clinical trial
Source: J Clin Microbiol. 2026 Apr 23;64(5):e01500-25. doi: 10.1128/jcm.01500-25 (PMC13170172; doi:10.1128/jcm.01500-25)
Supplement: Supplemental material — Tables S1 to S7; Fig. S1 to S3. [file jcm.01500-25-s0001.docx]

**Supplement for Manuscript:**

**Diagnostic performance of β-(1⭢3)-D-glucan, two *Candida*** **antigen and five anti-*Candida* antibody assays in ICU patients with sepsis and high risk for invasive candidiasis: a secondary end-point of the CandiSep randomized clinical trial**

**Supplementary Table 1: Median biomarker levels in patients with** **and without invasive *Candida* infection and candidemia**

| **biomarker** | **manufacturer cut-off value** | **no ICI** | **ICI (with candidemia)** | **p-value** | **ICI (w/o candidemia)** | **candidemia** | **p-value** |
| --- | --- | --- | --- | --- | --- | --- | --- |
| **BDG [pg/ml]**  (+/- IQR) | negative < 60  positive ≥ 80 | 62  (30-193) | 192  (38-640) | **0.003** | 106  (34-488) | 585  (52-1505) | **0.05** |
| **Platelia-Mn [pg/ml]**  (+/- IQR) | negative < 62.5  positive ≥ 125 | 20.0  (7.0-41.0) | 33.0  (13.0-160.0) | **0.001** | 27.0  (12.0-72.5) | 120.0  (26.3-406.3) | **0.06** |
| **Serion-Mn [U/ml]**  (+/- IQR) | negative < 1.4  positive > 2.6 | 0.6  (0.5-0.7) | 0.6  (0.5-1.1) | **0.022** | 0.6  (0.5-0.9) | 0.7  (0.6-2.1) | 0.30 |
| **Platelia-Ab [AU/ml]**  (+/- IQR) | negative < 5  positive ≥ 10 | 4.7  (2.0-9.5) | 5.5  (1.7-12.1) | 0.483 | 4.6  (1.3-8.4) | 5.5  (3.8-35.3) | 0.15 |
| **Serion-IgM [U/ml]** (+/- IQR) | negative < 60  positive > 80 | 6.0  (2.0-15.0) | 7.0  (3.0-19.0) | 0.338 | 10.0  (2.5-18.5) | 5.5  (3.5-29.0) | 0.94 |
| **Serion-IgG [U/ml]**  (+/- IQR) | negative < 40  positive > 100 | 15.0  (5.0-42.0) | 11.0  (3.0-45.0) | 0.882 | 9.0  (3.0-39.5) | 18.0  (6.8-158.3) | 0.13 |
| **Serion-IgA [U/ml]**  (+/- IQR) | negative < 60  positive > 80 | 5.0  (1.0-13.0) | 7.0  (2.0-18.0) | 0.315 | 7.0  (1.5-20.5) | 5.5  (2.0-15.0) | 0.968 |
| **CAGTA [Index]**  (+/- IQR) | negative < 0.9  positive > 1.1 | 0.3  (0-0.9) | 0.2  (0-1.0) | 0.898 | 0.2  (0-0.8) | 0.4  (0.1-2.8) | 0.16 |

p-values below 0.08 are shown in bold. BDG, β-(1⭢3)-D-glucan; IQR, interquartile range; Mn, mannan; U, units; Ab, antibody;
AU, arbitrary units; CAGTA, *Candida albicans* germ tube antibodies; ICI, invasive *Candida* infection; w/o, without.

**Supplementary Table 2: Median biomarker levels in patients with and without *Candida* colonization**

| **biomarker** | **ICI (with candidemia)** | ***Candida* colonization** | **no *Candida* colonization** | **p-value*** |
| --- | --- | --- | --- | --- |
| **BDG [pg/ml]**  (+/- IQR) | 171  (69-386) | 61  (30-286) | 66  (30-203) | 0.95 |
| **Platelia-Mn [pg/ml]**  (+/- IQR) | 34.0  (20.7-64.0) | 22.0  (8.0-48.0) | 21.0  (7.0-35.0) | 0.79 |
| **Serion-Mn [U/ml]**  (+/- IQR) | 0.6  (0.6-0.8) | 0.6  (0.5-0.7) | 0.5  (0.5-0.7) | 0.70 |
| **Platelia-Ab [AU/ml]**  (+/- IQR) | 5.5  (3.8-7.6) | 5.7  (2.6-11.4) | 2.8  (1.1-7.7) | **<0.001** |
| **Serion-IgM [U/ml]**  (+/- IQR) | 7.0  (5.0-12.7) | 8.0  (3.0-17.5) | 4.0  (1.0-12.5) | **<0.001** |
| **Serion-IgG [U/ml]**  (+/- IQR) | 11.0  (7.0-32.7) | 18.0  (5.0-46.5) | 11.5  (3.0-28.5) | **<0.001** |
| **Serion-IgA [U/ml]**  (+/- IQR) | 7.0  (3.0-11.4) | 6.0  (2.0-16.0) | 2.0  (0.0-9.0) | **<0.001** |
| **CAGTA [Index]**  (+/- IQR) | 0.2  (0.1-0.4) | 0.4  (0.1-1.1) | 0.1  (0.0-0.6) | **0.002** |

*p-value for comparison between patients with and without *Candida* colonization. p-values below 0.05 are shown in bold. ICI, invasive
*Candida* infection; BDG, β-(1⭢3)-D-glucan; IQR, interquartile range; Mn, mannan; U, units; Ab, antibody; AU, arbitrary units; CAGTA,
*Candida albicans* germ tube antibodies.

**Supplementary Table 3: Positive and negative predictive values of biomarker assays for different disease prevalences**

|  | **bio-**  **marker** | **invasive *Candida* infection (including candidemia)** | | | | | | | | **candidemia** | | | | | | | |
| --- | --- | --- | --- | --- | --- | --- | --- | --- | --- | --- | --- | --- | --- | --- | --- | --- | --- |
|  |  | **5% prevalence** | | **10% prevalence** | | **14% prevalence** | | **20% prevalence** | | **2% prevalence** | | **4.1% prevalence** | | **10% prevalence** | | **15% prevalence** | |
|  |  | **PPV [%]**  **(95%-CI)** | **NPV [%]**  **(95%-CI)** | **PPV [%]**  **(95%-CI)** | **NPV [%]**  **(95%-CI)** | **PPV [%]**  **(95%-CI)** | **NPV [%]**  **(95%-CI)** | **PPV [%]**  **(95%-CI)** | **NPV [%]**  **(95%-CI)** | **PPV [%]**  **(95%-CI)** | **NPV [%]**  **(95%-CI)** | **PPV [%]**  **(95%-CI)** | **NPV [%]**  **(95%-CI)** | **PPV [%]**  **(95%-CI)** | **NPV [%]**  **(95%-CI)** | **PPV [%]**  **(95%-CI)** | **NPV [%]**  **(95%-CI)** |
| **manu-facturers‘**  **cut-off**  **values** | **BDG** | 6.2  (4.9-7.9) | 96.1  (94.5-97.3) | 12.2  (9.7-15.3) | 92.2  (89.1-94.5) | 17.2  (13.8-21.2) | 88.8  (84.6-92.0) | 23.9  (19.5-28.9) | 84.0  (78.4-88.3) | 2.9  (2.1-4.1) | 98.9  (97.4-99.5) | 5.9  (4.2-8.1) | 97.7  (94.8-99.0) | 14.0  (10.3-18.7) | 94.1  (87.5-97.4) | 20.5  (15.4-26.8) | 91.0  (81.5-95.9) |
|  | **Platelia-Mn** | **13.8**  **(8.9-20.8)** | 96.3  (95.5-97.0) | **25.2**  **(17.0-35.6)** | 92.5  (90.9-93.8) | **33.4**  **(23.4-45.2)** | 89.2  (87.0-91.1) | **43.1**  **(31.6-55.4)** | 84.5  (81.5-87.1) | **7.0**  **(4.0-12.0)** | 98.8  (98.0-99.3) | **13.6**  **(8.0-22.2)** | 97.6  (96.0-98.6) | **29.1**  **(18.5-42.6)** | 94.0  (90.2-96.3) | **48.0**  **(33.8-62.5)** | 87.4  (80.4-92.1) |
|  | **Serion-Mn** | **18.3**  **(8.2-35.8)** | 95.6  (95.0-96.0) | **32.0**  **(15.9-54.1)** | 91.1  (90.0-92.0) | **41.3**  **(21.9-63.7)** | 87.2  (85.8-88.5) | **51.5**  **(29.8-72.6)** | 81.9  (80.1-83.6) | **12.7**  **(5.2-28.1)** | 98.5  (97.9-98.9) | **23.4**  **(10.2-45.0)** | 96.9  (95.8-97.8) | **44.2**  **(22.9-68.0)** | 92.4  (89.7-94.4) | **55.8**  **(32.0-77.1)** | 88.4  (84.5-91.4) |
|  | **Platelia-Ab** | 5.8  (3.6-9.0) | 95.3  (94.3-96.1) | 11.4  (7.4-17.3) | 90.5  (88.7-92.0) | 16.1  (10.6-23.8) | 86.5  (84.0-88.6) | 22.5  (15.2-32.0) | 80.9  (77.7-83.7) | 3.4  (1.8-6.2) | 98.5  (97.6-99.0) | 6.8  (3.8-12.2) | 96.8  (95.1-98.0) | 16.0  (9.2-26.5) | 92.2  (88.2-94.9) | 23.3  (13.9-36.8) | 88.2  (82.5-92.2) |
|  | **Serion-IgM** | **9.8**  **(3.0-28.0)** | 95.2  (94.8-95.5) | **18.7**  **(6.1-45.0)** | 90.3  (89.6-91.0) | **25.6**  **(8.8-55.0)** | 86.2  (85.2-87.2) | **34.1**  **(12.7-64.8)** | 80.6  (79.3-81.8) | **14.0**  **(4.8-34.8)** | 98.4  (97.9-98.8) | **25.5**  **(9.5-52.8)** | 96.7  (95.6-97.5) | **47.1**  **(21.4-74.4)** | 91.9  (89.4-93.9) | **58.5**  **(30.2-82.2)** | 87.7  (84.1-90.6) |
|  | **Serion-**  **IgG** | 5.8  (2.7-12.2) | 95.1  (94.5-95.6) | 11.5  (5.5-22.7) | 90.2  (89.1-91.2) | 16.3  (7.9-30.5) | 86.1  (84.5-87.5) | 22.7  (11.5-39.8) | 80.4  (78.4-82.2) | 7.0  (3.4-13.8) | 98.6  (97.9-99.1) | 13.5  (6.8-25.1) | 97.1  (95.7-98.1) | 28.9  (16.0-46.5) | 92.9  (89.5-95.3) | 39.2  (23.2-58.0) | 89.2  (84.3-92.7) |
|  | **Serion-**  **IgA** | 5.7  (1.4-20.7) | 95.0  (94.7-95.3) | 11.2  (2.8-35.6) | 90.1  (89.4-90.6) | 15.9  (4.1-45.1) | 85.9  (85.0-86.7) | 22.2  (6.1-55.4) | 80.1  (79.0-81.1) | 3.9  (0.6-22.6) | 98.1  (97.8-98.4) | 7.9  (1.2-38.0) | 96.1  (95.4-96.6) | 18.3  (3.0-61.4) | 90.4  (88.9-91.7) | 26.2  (4.8-71.7) | 85.5  (83.4-87.4) |
|  | **CAGTA** | 6.3  (3.5-11.0) | 95.3  (75.9-84.6) | 12.4  (7.2-20.7) | 90.5  (89.1-91.7) | 14.9  (9.1-23.5) | 86.0  (83.9-87.9) | 24.2  (14.8-37.0) | 80.9  (78.4-83.1) | 3.3  (1.6-6.7) | 98.4  (97.6-98.9) | 6.7  (3.3-13.0) | 96.6  (95.1-97.7) | 15.7  (8.2-28.0) | 91.7  (88.1-94.2) | 22.9  (12.5-38.2) | 87.4  (82.4-91.2) |
| **optimized**  **cut-off**  **values** | **BDG** | **11.0**  **(7.7-15.3)** | 96.6  (95.6-97.4) | **20.6**  **(15.0-27.7)** | 93.0  (91.1-94.6) | **27.8**  **(20.8-36.2)** | 90.0  (87.3-92.2) | **36.9**  **(28.5-46.2)** | 85.6  (82.0-88.6) | **5.7**  **(3.8-8.7)** | 99.1  (98.2-99.5) | **11.4**  **(7.6-16.7)** | 98.1  (96.2-99.0) | **24.9**  **(17.6-34.0)** | 95.2  (90.7-97.6) | **34.5**  **(25.3-45.0)** | 92.6  (86.0-96.2) |
|  | **Platelia-Mn** | **16.7**  **(10.6-25.3)** | 96.4  (95.6-97.1) | **29.8**  **(20.1-41.7)** | 92.7  (91.1-94.0) | **38.6**  **(27.2-51.5)** | 89.5  (87.3-91.3) | **48.8**  **(36.1-61.7)** | 84.9  (82.0-87.4) | **10.9**  **(6.9-16.7)** | 99.2  (98.4-99.6) | **20.5**  **(13.5-29.8)** | 98.3  (96.6-99.2) | **39.9**  **(28.7-52.2)** | 95.7  (91.8-97.8) | **51.3**  **(39.0-63.5)** | 93.4  (87.5-96.6) |
|  | **Serion-Mn** | **17.54**  **(9.9-29.2)** | 96.0  (95.3-96.6) | **31.0**  **(18.8-46.6)** | 91.8  (90.5-93.0) | **40.0**  **(25.6-56.4)** | 88.4  (86.5-90.0) | **50.3**  **(34.2-66.2)** | 83.3  (80.9-85.5) | **8.7**  **(4.1-17.4)** | 98.6  (98.0-99.0) | **16.7**  **(8.3-30.7)** | 97.1  (95.8-98.0) | **34.0**  **(18.9-53.4)** | 92.8  (89.7-95.0) | **45.0**  **(27.0-64.5)** | 89.1  (84.6-92.3) |
|  | **Platelia-Ab** | 5.7  (5.4-6.0) | 99.2  (94.9-99.9) | 11.3  (10.7-11.9) | 98.4  (89.8-99.8) | 15.9  (15.1-16.7) | 97.7  (85.5-99.7) | 22.2  (21.2-23.3) | 96.5  (79.6-99.5) | 2.1  (1.8-2.5) | 98.9  (92.9-99.8) | 4.4  (3.8-5.1) | 97.7  (86.2-99.6) | 10.6  (9.2-12.1) | 94.2  (70.7-99.1) | 15.8  (13.9-18.0) | 91.1  (60.3-98.6) |
|  | **Serion-IgM** | 6.3  (4.6-8.7) | 95.8  (94.5-96.8) | 12.5  (9.2-16.7) | 91.5  (89.0-93.4) | 17.2  (12.9-22.6) | 88.1  (84.8-90.7) | 24.3  (18.6-31.1) | 82.7  (78.3-86.4) | 1.9  (0.9-3.8) | 97.9  (96.9-98.6) | 3.9  (1.9-7.7) | 95.7  (93.7-97.1) | 9.5  (4.9-17.6) | 89.7  (85.3-92.8) | 14.2  (7.5-25.4) | 84.5  (78.6-89.1) |
|  | **Serion-**  **IgG** | 4.4  (3.5-5.5) | 93.7  (90.8-95.7) | 8.9  (7.2-11.0) | 87.5  (82.4-91.3) | 12.4  (10.1-15.3) | 82.9  (76.4-87.9) | 18.0  (14.8-21.8) | 75.7  (67.6-82.4) | 1.7  (1.1-2.6) | 97.2  (94.9-98.5) | 3.4  (2.2-5.3) | 94.3  (89.8-96.9) | 8.4  (5.5-12.6) | 86.5  (77.4-92.3) | 12.7  (8.4-18.7) | 80.2  (68.3-88.3) |
|  | **Serion-**  **IgA** | 6.3  (4.7-8.3) | 95.9  (94.5-97.0) | 12.4  (9.5-16.0) | 91.8  (89.0-93.9) | 17.0  (13.2-21.7) | 88.5  (84.8-91.4) | 24.1  (19.0-30.0) | 83.2  (78.3-87.2) | 2.3  (1.4-3.9) | 98.2  (97.0-99.0) | 4.8  (2.8-7.9) | 96.3  (93.9-97.8) | 11.4  (7.0-18.1) | 91.1  (85.7-94.6) | 17.0  (10.6-25.9) | 86.6  (79.1-91.6) |
|  | **CAGTA** | 5.5  (4.5-6.8) | 95.8  (93.7-97.2) | 11.0  (9.0-13.3) | 91.5  (87.6-94.3) | 15.5  (12.8-18.6) | 87.9  (82.6-91.7) | 21.7  (18.2-25.7) | 82.7  (75.8-88.0) | 1.9  (1.2-2.9) | 97.8  (96.0-98.8) | 3.9  (2.5-6.0) | 95.5  (91.9-97.5) | 9.4  (6.1-14.1) | 89.1  (81.4-93.8) | 14.1  (9.4-20.7) | 83.7  (73.4-90.5) |

In order to assess the diagnostic performance of the biomarkers in other patient cohorts, the PPV and NPV are given for different prevalences. The PPV and NPV for a prevalence of 14.0% (ICI) and 4.1% (candidemia) correspond to those in the present study. The three best PPV results are highlighted in bold. BDG, β-(1⭢3)-D-glucan; Mn, mannan; Ab, antibody; CAGTA, *Candida albicans* germ tube antibodies; CI, confidence interval; PPV, positive predictive value; NPV, negative predictive value.

**Supplementary Table 4:** **Diagnostic performance of biomarkers for diagnosis of ICI in patients with and without *Candida* colonization**

|  | **biomarker** | **non-*Candida*-colonized patients** | | | ***Candida*-colonized patients** | | |
| --- | --- | --- | --- | --- | --- | --- | --- |
|  |  | **sensitivity [%]**  **(95%-CI)** | **specificity [%]**  **(95%-CI)** | **AUROC [%]**  **(95%-CI)** | **sensitivity [%]**  **(95%-CI)** | **specificity [%]**  **(95%-CI)** | **AUROC [%]**  **(95%-CI)** |
| **manufacturers‘**  **cut-off**  **values** | **BDG** | **57.1**  **(28.9-82.3)** | 57.9  (47.3-68.0) | **0.575**  **(0.477-0.669)** | **61.8**  **(43.6-77.8)** | 49.0  (41.8-56.2) | **0.554**  **(0.487-0.619)** |
|  | **Platelia-Mn** | **35.7**  **(12.8-64.9)** | 89.5  (81.5-94.8) | **0.626**  **(0.528-0.717)** | **35.3**  **(19.7-53.5)** | 87.8  (82.3-92.0) | **0.615**  **(0.549-0.678)** |
|  | **Serion-Mn** | 0.0  (0.0-23.2) | **95.8**  **(89.6-98.8)** | 0.479  (0.382-0.577) | 20.6  (8.7-37.9) | **96.9**  **(93.5-98.9)** | **0.588**  **(0.521-0.652)** |
|  | **Platelia-Ab** | 21.4  (4.7-50.8) | 74.7  (64.8-83.1) | 0.481  (0.384-0.579) | **32.4**  **(17.9-50.5)** | 75.0  (68.3-80.9) | 0.537  (0.470-0.603) |
|  | **Serion-IgM** | 0.0  (0.0-24.7) | **94.7**  **(88.0-98.3)** | 0.473  (0.376-0.572) | 9.4  (2.0-25.0) | **97.8**  **(94.6-99.4)** | 0.536  (0.468-0.604) |
|  | **Serion-IgG** | 0.0  (0.0-24.7) | 88.3  (80.0-94.0) | 0.441  (0.346-0.541) | 18.8  (7.2-36.4) | 88.8  (83.4-93.0) | 0.538  (0.470-0.605) |
|  | **Serion-IgA** | 7.7  (0.2-36.0) | **95.7**  **(89.5-98.8)** | 0.517  (0.419-0.615) | 3.1  (0.1-16.2) | **96.3**  **(92.4-98.5)** | 0.497  (0.429-0.565) |
|  | **CAGTA** | **28.6**  **(8.4-58.1)** | 75.8  (65.9-84.0) | **0.522**  **(0.424-0.618)** | 20.6  (8.7-37.9) | 79.6  (73.3-85.0) | 0.501  (0.434-0.567) |
| **optimized**  **cut-off**  **values** | **BDG** | 35.7  (12.8-64.9) | **81.1**  **(71.7-88.4)** | **0.584**  **(0.485-0.677)** | 50.0  (32.4-67.6) | **80.1**  **(73.8-85.5)** | **0.651**  **(0.585-0.712)** |
|  | **Platelia-Mn** | 21.4  (4.7-50.8) | **90.5**  **(82.8-95.6)** | 0.560  (0.461-0.655) | 41.2  (24.6-59.3) | **90.8**  **(85.9-94.5)** | **0.660**  **(0.595-0.721)** |
|  | **Serion-Mn** | 14.3  (1.8-42.8) | **92.6**  **(85.4-97.0)** | 0.535  (0.437-0.631) | 29.4  (15.1-47.5) | **94.4**  **(90.2-97.2)** | **0.619**  **(0.553-0.682)** |
|  | **Platelia-Ab** | **92.9**  **(66.1-99.8)** | 12.6  (6.7-21.0) | 0.527  (0.430-0.624) | **100.0**  **(89.7-100.0)** | 15.3  (10.6-21.1) | 0.577  (0.510-0.641) |
|  | **Serion-IgM** | 50.0  (23.0-77.0) | 68.4  (58.1-77.6) | **0.592**  **(0.494-0.685)** | 45.5  (28.1-63.6) | 61.2  (54.0-68.1) | 0.533  (0.467-0.599) |
|  | **Serion-IgG** | **64.3**  **(35.1-87.2)** | 27.4  (18.7-37.5) | 0.458  (0.362-0.556) | **60.6**  **(42.1-77.1)** | 31.1  (24.7-38.1) | 0.459  (0.393-0.526) |
|  | **Serion-IgA** | **57.1**  **(28.9-82.3)** | 62.1  (51.6-71.9) | **0.596**  **(0.498-0.689)** | 51.5  (33.5-69.2) | 56.1  (48.9-63.2) | 0.538  (0.471-0.604) |
|  | **CAGTA** | **57.1**  **(28.9-82.3)** | 40.0  (30.1-50.6) | 0.486  (0.389-0.583) | **70.6**  **(52.5-84.9)** | 39.8  (32.9-47.0) | 0.552  (0.485-0.617) |

BDG, β-(1⭢3)-D-glucan; Mn, mannan; Ab, antibody; CAGTA, *Candida albicans* germ tube antibodies; CI, confidence interval; AUROC, area under the ROC curve.

**Supplementary Table 5: Diagnostic performance of biomarkers** **in patients with and without recent abdominal surgery**

|  | **biomarker** | **no recent abdominal surgery** | | | **recent abdominal surgery** | | |
| --- | --- | --- | --- | --- | --- | --- | --- |
|  |  | **sensitivity [%]**  **(95%-CI)** | **specificity [%]**  **(95%-CI)** | **AUROC [%]**  **(95%-CI)** | **sensitivity [%]**  **(95%-CI)** | **specificity [%]**  **(95%-CI)** | **AUROC [%]**  **(95%-CI)** |
| **manufacturers‘**  **cut-off**  **values** | **BDG** | **50,0**  **21,1-78,9** | 56,4  45,8-66,6 | **0,532**  **0,432-0,630** | **63,9**  **46,2-79,2** | 49,7  42,6-56,9 | **0,568**  **0,502-0,633** |
|  | **Platelia-Mn** | **33,3**  **9,9-65,1** | **94,7**  **88,0-98,3** | **0,640**  **0,541-0,731** | **36,1**  **20,8-53,8** | 85,3  79,5-89,9 | **0,607**  **0,541-0,670** |
|  | **Serion-Mn** | 16,7  2,1-48,4 | **97,9**  **92,5-99,7** | **0,573**  **0,473-0,668** | 13,9  4,7-29,5 | **95,9**  **92,2-98,2** | **0,549**  **0,483-0,614** |
|  | **Platelia-Ab** | 16,7  2,1-48,4 | 74,5  64,4-82,9 | 0,456  0,359-0,555 | **33,3**  **18,6-51,0** | 75,1  68,5-81,0 | 0,542  0,476-0,608 |
|  | **Serion-IgM** | 9,1  0,2-41,3 | **97,8**  **92,1-99,7** | **0,534**  **0,432-0,635** | 5,9  0,7-19,7 | **96,3**  **92,6-98,5** | 0,511  0,444-0,578 |
|  | **Serion-IgG** | 9,1  0,2-41,3 | 85,6  76,6-92,1 | 0,473  0,373-0,575 | 14,7  5,0-31,1 | 90,1  85,093,9 | 0,524  0,457-0,591 |
|  | **Serion-IgA** | 0,0  0,0-28,5 | 95,5  88,9-98,8 | 0,478  0,377-0,580 | 5,9  0,7-19,7 | **96,4**  **92,6-98,5** | 0,511  0,444-0,578 |
|  | **CAGTA** | **25,0**  **5,5-57,2** | 73,4  63,3-82,0 | 0,492  0,394-0,591 | 22,2  10,1-39,2 | 80,7  74,5-86,0 | 0,515  0,449-0,580 |
| **optimized**  **cut-off**  **values** | **BDG** | 41,7  15,2-72,3 | **75,5**  **65,6-83,8** | **0,586**  **0,486-0,681** | 47,2  30,4-64,5 | **82,7**  **76,7-87,7** | **0,650**  **0,585-0,711** |
|  | **Platelia-Mn** | 25,0  5,5-57,2 | **94,7**  **88,0-98,3** | **0,598**  **0,499-0,692** | 38,9  23,1-56,5 | **88,8**  **83,6-92,9** | **0,639**  **0,573-0,700** |
|  | **Serion-Mn** | 16,7  2,1-48,4 | **96,8**  **91,0-99,3** | **0,567**  **0,468-0,663** | 27,8  14,2-45,2 | **92,4**  **87,8-95,7** | **0,601**  **0,535-0,664** |
|  | **Platelia-Ab** | **91,7**  **61,5-99,8** | 8,5  3,7-16,1 | 0,501  0,402-0,600 | **100,0**  **90,3-100,0** | 17,3  12,3-23,3 | 0,586  0,5200,650 |
|  | **Serion-IgM** | 36,4  10,9-69,2 | 61,7  51,1-71,5 | 0,490  0,391-0,590 | 50,0  32,9-67,1 | 64,5  57,4-71,1 | 0,572  0,506-0,637 |
|  | **Serion-IgG** | **63,6**  **30,8-89,1** | 31,9  22,7-42,3 | 0,478  0,379-0,577 | **61,1**  **43,5-76,9** | 28,9  22,7-35,8 | 0,450  0,385-0,517 |
|  | **Serion-IgA** | 36,4  10,9-69,2 | 54,3  43,7-64,6 | 0,453  0,356-0,553 | 58,3  40,8-74,5 | 59,9  52,7-66,8 | 0,591  0,5250,655 |
|  | **CAGTA** | **75,0**  **42,8-94,5** | 39,4  29,4-50,0 | **0,572**  **0,472-0,667** | **63,9**  **46,2-79,2** | 40,1  33,2-47,3 | 0,520  0,454-0,586 |

BDG, β-(1⭢3)-D-glucan; Mn, mannan; Ab, antibody; CAGTA, *Candida albicans* germ tube antibodies; CI, confidence interval; AUROC, area under the ROC curve.

**Supplementary Table 6: Diagnostic performance of assay combinations at the manufacturers’ cut-off values for the measurement of the first serum sample**

|  | **invasive Candida infection (including candidemia)** | | | | | **candidemia** | | | | |
| --- | --- | --- | --- | --- | --- | --- | --- | --- | --- | --- |
|  | **sensitivity [%]**  **(95%-CI)** | **specificity [%]**  **(95%-CI)** | **PPV [%]**  **(95%-CI)** | **NPV [%]**  **(95%-CI)** | **AUROC [%]**  **(95%-CI)** | **sensitivity [%]**  **(95%-CI)** | **specificity [%]**  **(95%-CI)** | **PPV [%]**  **(95%-CI)** | **NPV [%]**  **(95%-CI)** | **AUROC [%]**  **(95%-CI)** |
| **BDG +**  **Platelia-Mn** | 70.8  (55.9-83.0) | 47.8  (41.9-53.7) | 18.3  (15.4-21.7) | 90.8  (86.2-94.0) | 0.593  (0.539-0.646) | 85.7  (57.2-98.2) | 46.5  (40.9-52.0) | 6.4  (5.1-8.0) | 98.7  (95.4-99.6) | 0.661  (0.608-0.711) |
| **BDG +**  **Serion-Mn** | 60.4  (45.3-74.2) | 50.9  (45.0-56.7) | 16.9  (13.6-20.8) | 88.6  (84.3-91.8) | 0.556  (0.502-0.610) | 71.4  (41.9-91.6) | 50.2  (44.6-55.7) | 5.8  (4.1-8.0) | 97.6  (94.7-99.6) | 0.608  (0.554-0.660) |
| **BDG +**  **Platelia-Ab** | 66.7  (51.6-79.6) | 40.2  (34.5-46.1) | 15.6  (12.9-18.7) | 87.9  (82.7-91.8) | 0.534  (0.480-0.588) | 85.7  (57.2-98.2) | 40.3  (34.9-45.9) | 5.8  (4.6-7.2) | 98.5  (94.9-99.6) | 0.630  (0.576-0.682) |
| **BDG +**  **Serion-IgA/IgG/IgM** | 66.7  (51.6-79.6) | 46.4  (40.6-52.3) | 17.1  (14.1-20.5) | 89.4  (84.7-92.7) | 0.565  (0.511-0.619) | 85.7  (57.2-98.2) | 45.8  (40.3-51.4) | 6.3  (5.1-7.9) | 98.7  (95.4-99.6) | 0.658  (0.605-0.708) |
| **BDG +**  **CAGTA** | 68.8  (53.7-81.3) | 40.6  (34.9-46.4) | 16.1  (13.4-19.1) | 88.7  (83.4-92.4) | 0.546  (0.492-0.600) | 85.7  (57.2-98.2) | 40.3  (34.9-45.9) | 5.8  (4.6-7.2) | 98.5  (94.8-99.6) | 0.630  (0.576-0.682) |
| **Platelia-Mn +**  **Serion-Mn** | 35.4  (22.2-50.5) | 87.3  (82.9-90.9) | 31.6  (22.1-42.9) | 89.1  (86.8-91.0) | 0.614  (0.559-0.666) | 50.0  (23.0-77.0) | 85.5  (81.2-89.2) | 12.9  (7.6-21.0) | 97.6  (95.9-98.5) | 0.678  (0.625-0.727) |
| **Platelia-Mn +**  **Platelia-Ab** | 56.3  (41.2-70.5) | 65.3  (59.5-70.8) | 21.1  (16.6-26.5) | 90.0  (86.6-92.6) | 0.608  (0.554-0.660) | 78.6  (49.2-95.3) | 64.0  (58.5-69.2) | 8.5  (6.4-11.3) | 98.6  (96.2-99.5) | 0.713  (0.662-0.760) |
| **Platelia-Mn +**  **Serion-IgA/IgG/IgM** | 43.8  (29.5-58.8) | 76.3  (71.0-81.1) | 23.4  (17.3-30.9) | 89.1  (86.4-91.4) | 0.600  (0.546-0.653) | 71.4  (41.9-91.6) | 75.4  (70.3-80.0) | 11.0  (7.8-15.4) | 98.4  (96.4-99.3) | 0.734  (0.684-0.780) |
| **Platelia-Mn +**  **CAGTA** | 52.1  (37.2-66.7) | 68.0  (62.3-73.4) | 21.2  (16.4-27.1) | 89.6  (86.3-92.1) | 0.601  (0.546-0.653) | 71.4  (41.9-91.6) | 66.8  (61.4-71.9) | 8.4  (6.0-11.7) | 98.2  (96.0-99.2) | 0.691  (0.639-0.740) |
| **Serion-Mn +**  **Platelia-Ab** | 43.8  (29.5-58.8) | 71.5  (65.9-76.6) | 20.2  (14.9-26.9) | 88.5  (85.6-90.9) | 0.576  (0.522-0.629) | 71.4  (41.9-91.6) | 71.1  (65.8-75.9) | 9.6  (6.8-13.3) | 98.3  (96.2-99.3) | 0.713  (0.661-0.760) |
| **Serion-Mn +**  **Serion-IgA/IgG/IgM** | 29.2  (17.0-44.1) | 83.8  (79.1-87.9) | 23.0  (15.1-33.3) | 87.7  (85.6-89.6) | 0.565  (0.510-0.619) | 64.3  (35.1-87.2) | 84.0  (79.6-87.8) | 14.7  (9.8-21.4) | 98.2  (96.5-99.1) | 0.741  (0.691-0.787) |
| **Serion-Mn +**  **CAGTA** | 37.5  (24.0-52.6) | 74.6  (69.2-79.5) | 19.6  (13.9-27.0) | 87.8  (85.1-90.1) | 0.560  (0.506-0.614) | 64.3  (35.1-87.2) | 74.5  (69.4-79.1) | 9.7  (6.5-14.2) | 98.0  (96.0-99.0) | 0.694  (0.642-0.742) |

A combination was considered positive if at least one assay from one serum was positive. BDG combinations had the highest sensitivity (60.4-85.7%) and the lowest specificity (40.2-50.9%), regardless of the additional assay. The highest AUROC values in ICI were achieved by Platelia-Mn combinations and in candidemia by Platelia-Mn combinations and Serion-Mn combinations. BDG, β-(1⭢3)-D-glucan; Mn, mannan; Ab, antibody; CAGTA, *Candida albicans* germ tube antibodies; CI, confidence interval; PPV, positive predictive value; NPV, negative predictive value; AUROC, area under the ROC curve.

**Supplementary Table 7: Overview of prospective biomarker studies in ICU patients with invasive *Candida* infection**

| **first author** | **year** | **patients** | **patient groups (no. of patients)** | **BDG** | | **Platelia-Mn** | | **Serion-Mn** | | **Platelia-Ab** | | **Serion-**  **IgM/IgG/IgA** | | **CAGTA** | |
| --- | --- | --- | --- | --- | --- | --- | --- | --- | --- | --- | --- | --- | --- | --- | --- |
|  |  |  |  | **sens** | **spec** | **sens** | **spec** | **sens** | **spec** | **sens** | **spec** | **sens** | **spec** | **sens** | **spec** |
| Posteraro B | 2011 | ICU with sepsis | no IFD (79)  ICI (14) | 92.9 | 93.7 | --- | | --- | | --- | | --- | | --- | |
| Leon C | 2012 | ICU with SAC | not colonised, not infected (61);  colonised, not infected (84);  ICI (31) | 51.6^1^ | 86.9^1^ | --- | | --- | | --- | | --- | | 71.0^2^ | 57.3^2^ |
| Tissot F | 2013 | ICU with high risk for IAC | not colonised, not infected (2);  colonised, not infected (58);  ICI (29) | 83 | 40 | --- | | --- | | --- | | --- | | --- | |
| Martin-Mazuelos E | 2015 | ICU, unselected | not colonised, not infected (29);  colonised, not infected (63);  ICI (15) | 80.0 | 44.6 | --- | | --- | | --- | | --- | | 33.3 | 55.4 |
| Leon C | 2016 | ICU with SAC | not colonised, not infected (48);  colonised, not infected (154);  ICI (31) | 76.7 | 57.2 | 43.3^3^ | 67.3^3^ | --- | | 25.8 | 89.0 | --- | | 53.3^2^ | 64.3^2^ |
| Christner M | 2024 | ICU with suspected ICI | no ICI (128)  ICI (46) | 77^4^ | 32^4^ | --- | | --- | | --- | | --- | | --- | |
| Novy E | 2023 | ICU with risk for IAC and surgery | no ICI (112)  ICI (87) | 59.0 | 63.3 | --- | | --- | | --- | | --- | | --- | |
| Standl L | 2025 | ICU with sepsis and high risk for ICI | no ICI (291)  ICI (48) | 60.4^5^ | 51.9^5^ | 35.4^5^ | 88.3^5^ | 14.6^5^ | 96.6^5^ | 29.2^5^ | 74.9^5^ | 6.7^5^  13.3^5^  4.4^5^ | 96.8^5^  88.7^5^  96.1^5^ | 22.9^5^ | 78.4^5^ |
|  |  |  |  | 45.8^6^ | 80.4^6^ | 35.4^6^ | 88.3^6^ | 25.0^6^ | 93.8^6^ | 97.9^6^ | 14.4^6^ | 46.8^6^  61.7^6^  53.2^6^ | 63.6^6^  29.9^6^  58.1^6^ | 66.8^6^ | 39.9^6^ |

^1^ A BDG cut-off value of 260 pg/ml was used instead of 80 pg/ml. ^2^ The CAGTA immunofluorescence assay was used instead of the CAGTA-CLIA monotest. ^3^ A Platelia-Mn cut-off value of 60 pg/ml was used instead of 125 pg/ml. ^4^ This study used the Fujifilm Wako β-glucan test instead of the Fungitell assay. ^5^ Results for the manufacturers’ cut-off values. ^6^ Results for the optimized cut-off values. No., number; BDG, β-(1⭢3)-D-glucan; Mn, mannan; Ab, antibody; CAGTA, *Candida albicans* germ tube antibodies; sens, sensitivity in %; spec, specificity in %; ICU, intensive care unit; IFD, invasive fungal disease; ICI, invasive *Candida* infection; SAC, severe abdominal conditions; IAC, intra-abdominal candidiasis.

**Effect of storage in frozen state on biomarker values:**

In our study, the sera were tested for BDG between 09/2016 and 09/2019 and for the other biomarkers between 02/2019 and 12/2019. It is therefore possible that biomarker concentrations could have decreased during storage or because of freeze-thaw cycles.

After the BDG test, the sera were divided into aliquots for each additional biomarker, i.e., there was only one freeze-thaw cycle between the BDG test and the tests for the other biomarkers.

With regard to degradation during storage, several studies demonstrated the long-term stability of antibodies against various pathogens. (e.g. SARS-CoV-2, *Toxoplasma gondii*, adenovirus, hepatitis A and B virus; PMID: 28093212, 24958122, 40490397). Despite a lack of data on anti-mannan antibodies or CAGTA it seems unlikely that these antibodies should react differently.

For mannan antigen, we found no studies on the stability during storage. However, galactomannan stability was assessed in a number of studies with conflicting results. Wheat *et al.* reported galactomannan stability for at least 5 years at -20 °C (1). In contrast, Johnson *et al.* and others reported a decrease in galactomannan concentration over time (2-6), but came to conflicting conclusions as to whether this applied only to serum samples or also to BALF samples. Interestingly, two of the five studies reported that strongly decreasing galactomannan levels were present only in patients without probable invasive aspergillosis. In contrast, among patients with invasive aspergillosis, the second measurement was only marginally lower (<0,1 AI), suggesting that the observed decrease may not reflect a true decline in galactomannan but rather a false-positive result at the initial testing (5, 6).

Most of the sera of the CandiSep study were used up for biomarker testing. Only 3 sera from patients with candidemia could be re-tested with the following results:

| **No.** | **Date of original testing** | **Mannan concentration [pg/ml]** | **Date of re-testing** | **Mannan concentration [pg/ml]** | **Difference**  **[pg/ml]** | **Difference**  **[%]** |
| --- | --- | --- | --- | --- | --- | --- |
| 85a | 21.02.19 | 545 | 21.01.26 | 679 | +134 | +25 |
| 85b | 21.02.19 | 262 | 21.01.26 | 251 | -11 | -4 |
| 86a | 21.02.19 | 330 | 21.01.26 | 1311 | +981 | +297 |

No relevant mannan degradation was detected in any of the samples. Rather, it must be assumed that contamination occurred in two of the samples during further sample processing. In order to increase the number of samples for comparison, we tested 20 additional mannan positive samples from 2020 out of diagnostic routine:

| **no.** | **date of original testing** | **mannan concentration [pg/ml]** | **date of  re-testing** | **mannan concentration [pg/ml]** | **difference**  **[pg/ml]** | **difference**  **[%]** |
| --- | --- | --- | --- | --- | --- | --- |
| se02835 | 02.04.2020 | 131 | 01/2026 | 137 | 6 | 4.3 |
| se10586 | 11.02.2020 | 135 | 01/2026 | 33 | -102 | -75.3 |
| se10075 | 26.11.2020 | 135 | 01/2026 | 107 | -28 | -20.7 |
| se04099 | 18.05.2020 | 138 | 01/2026 | 155 | 17 | 12.6 |
| se08821 | 21.10.2020 | 139 | 01/2026 | 26 | -113 | -81.1 |
| se05448 | 01.07.2020 | 142 | 01/2026 | 135 | -7 | -4.8 |
| se01645 | 20.02.2020 | 143 | 01/2026 | 134 | -9 | -6.2 |
| se04975 | 17.06.2020 | 205 | 01/2026 | 55 | -150 | -73.4 |
| se01997 | 04.03.2020 | 212 | 01/2026 | 269 | 57 | 26.7 |
| se01424 | 14.02.2020 | 219 | 01/2026 | 248 | 29 | 13.1 |
| se09548 | 12.11.2020 | 222 | 01/2026 | 38 | -184 | -82.9 |
| se02786 | 01.04.2020 | 230 | 01/2026 | 255 | 25 | 11.0 |
| se03136 | 16.04.2020 | 516 | 01/2026 | 450 | -66 | -12.8 |
| se07977 | 25.09.2020 | 530 | 01/2026 | 419 | -111 | -21.0 |
| se01925 | 02.03.2020 | 656 | 01/2026 | 598 | -58 | -8.8 |
| se06414 | 04.05.2020 | 723 | 01/2026 | 525 | -198 | -27.4 |
| se02403 | 18.03.2020 | 748 | 01/2026 | 688 | -60 | -8.0 |
| se06065 | 22.07.2020 | 812 | 01/2026 | 938 | 126 | 15.5 |
| se05860 | 15.07.2020 | 860 | 01/2026 | 893 | 33 | 3.9 |
| se07716 | 17.09.2020 | 1087 | 01/2026 | 1024 | -63 | -5.8 |

A comparison of the medians using the Wilcoxon test showed that the mean mannan concentration was significantly higher in the measurement in 2020 (208 pg/ml, IQR 138-445) than in the second measurement 2025 (146 pg/ml, IQR 68-382; p=0.026). Surprisingly, some of the results in the second measurement were even completely negative, an observation that could be explained either by a complete degradation of the mannan or by a false-positive first measurement. All those negative sera were from patients without proven invasive candidosis and based on our data of the Candisep study, it seems clear that the Platelia mannan assay also causes false-positive results. In Candisep patients without ICI, 11 (58%) of 19 mannan-positive results in the serum from day 1 (154–9667 pg/ml) were completely negative in the serum from day 2 (0–34 pg/m). If those samples (se10586, se08821, se04975, se09548) are excluded from the analysis the medians of the measurement in 2020 and 2025 are not significantly different any more (median 2020: 216 pg/ml, IQR 139-527; median 2025: 252 pg/ml, IQR 136-442; p=0.272).

In summary, we cannot rule out the possibility that mannan degrades in frozen serum over time, but it seems equally likely that no degradation has occurred.


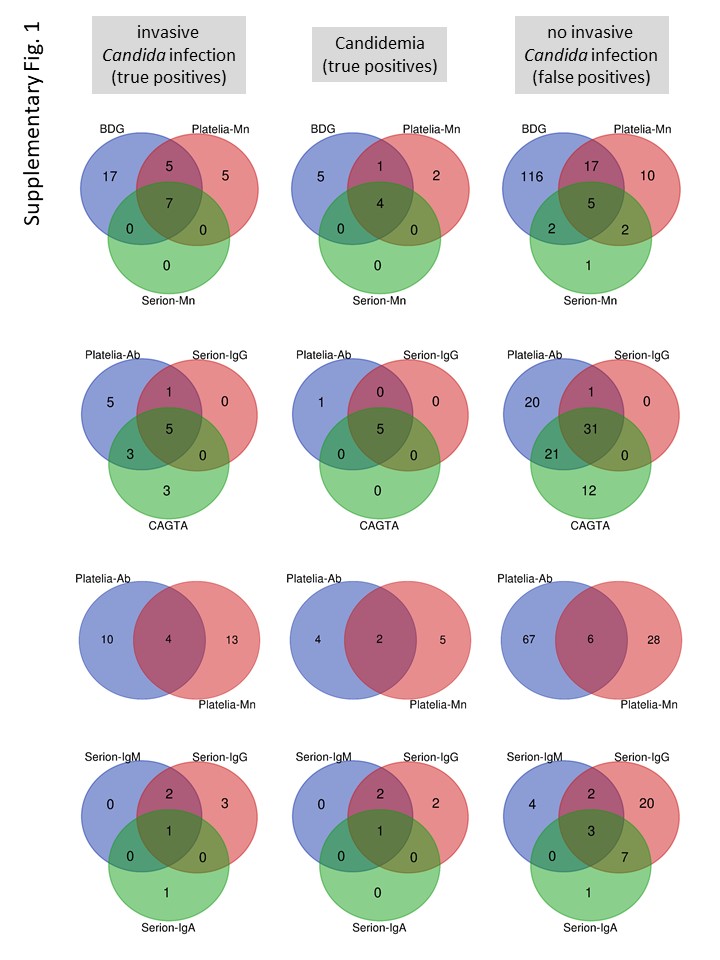


Venn diagrams showing the true positive and false positive test results at the manufacturers’ cut-off values. BDG, β-(1⭢3)-D-glucan; Mn, mannan; Ab, antibody; CAGTA, *Candida albicans* germ tube antibodies.


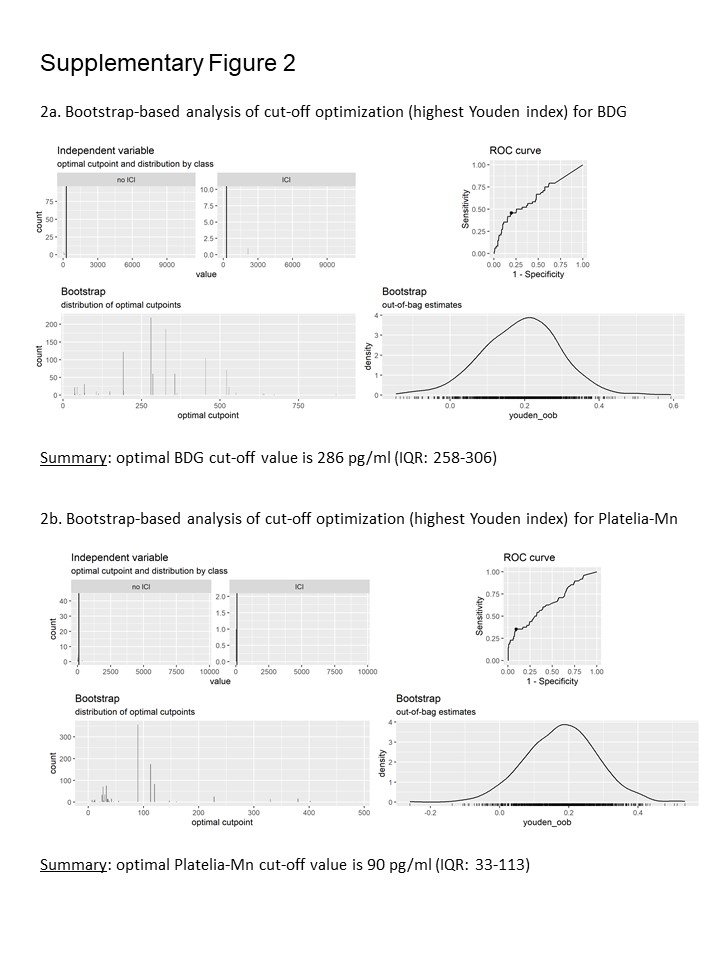


BDG, β-(1⭢3)-D-glucan; Mn, mannan; ICI, invasive *Candida* infection.


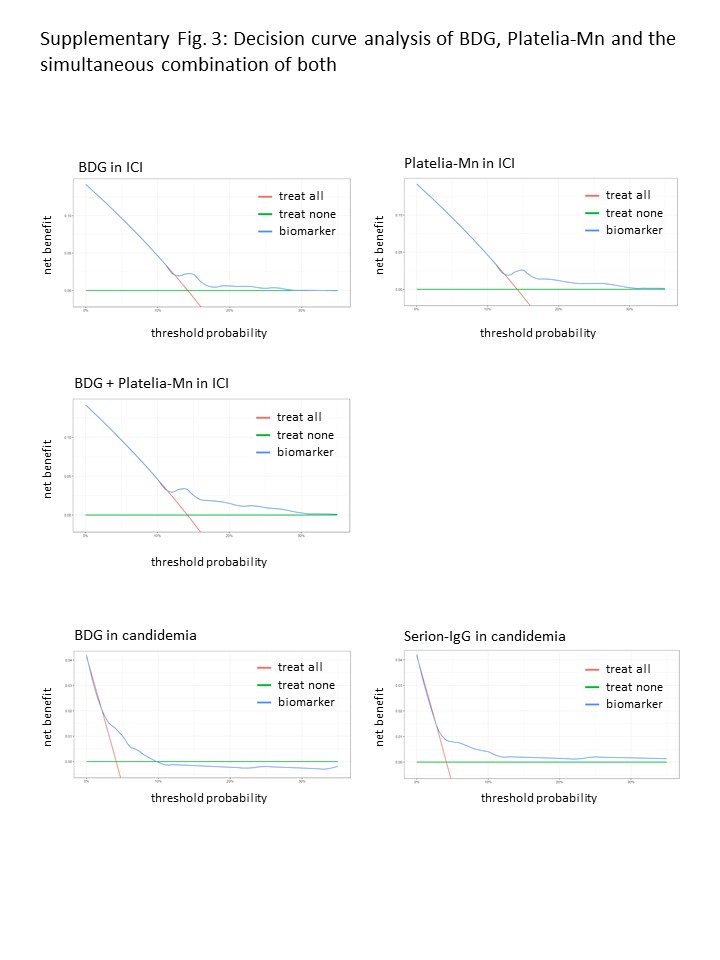


BDG, β-(1⭢3)-D-glucan; Mn, mannan; ICI, invasive *Candida* infection.

References:

1. Wheat LJ, Nguyen MH, Alexander BD, Denning D, Caliendo AM, Lyon GM, Baden LR, Marty FM, Clancy C, Kirsch E, Noth P, Witt J, Sugrue M, Wingard JR. 2014. Long-term stability at -20 degrees C of Aspergillus galactomannan in serum and bronchoalveolar lavage specimens. J Clin Microbiol 52:2108-11.

2. Johnson GL, Sarker SJ, Hill K, Tsitsikas DA, Morin A, Bustin SA, Agrawal SG. 2013. Significant decline in Galactomannan Signal during storage of clinical serum samples. Int J Mol Sci 14:12970-7.

3. Dufresne SF, Beauchemin S, Lavallee C, Laverdiere M. 2014. Instability of Aspergillus galactomannan in stored clinical samples. J Clin Microbiol 52:4435-6.

4. Oren I, Avidor I, Sprecher H. 2012. Lack of intra-laboratory reproducibility in using Platelia Aspergillus enzyme immunoassay test for detection of Aspergillus galactomannan antigen. Transpl Infect Dis 14:107-9.

5. Furfaro E, Mikulska M, Miletich F, Viscoli C. 2012. Galactomannan: testing the same sample twice? Transpl Infect Dis 14:E38-9.

6. Bizzini A, Marchetti O, Meylan P. 2012. Response to: lack of intra-laboratory reproducibility in using Platelia Aspergillus enzyme immunoassay test for detection of Aspergillus galactomannan antigen. Transpl Infect Dis 14:218-9.
